# Amplified antitumor efficacy by a targeted drug retention and chemosensitization strategy-based “combo” nanoagent together with PD-L1 blockade in reversing multidrug resistance

**DOI:** 10.1186/s12951-021-00947-9

**Published:** 2021-07-05

**Authors:** Weixi Jiang, Lei Su, Meng Ao, Xun Guo, Chen Cheng, Yuanli Luo, Zhuoyan Xie, Xingyue Wang, Junrui Wang, Shuling Liu, Yang Cao, Pan Li, Zhigang Wang, Haitao Ran, Zhiyi Zhou, Jianli Ren

**Affiliations:** 1 Department of Ultrasound, Chongqing Key Laboratory of Ultrasound Molecular Imaging, The Second Affiliated Hospital of Chongqing Medical University, Chongqing, 400010 People’s Republic of China; 2 Department of Radiology, The Second Affiliated Hospital of Chongqing Medical University, Chongqing, 400010 People’s Republic of China; 3 Department of General Practice of Chongqing General Hospital, University of Chinese Academy of Sciences, Chongqing, 401147 People’s Republic of China

**Keywords:** Multidrug resistance, Endo/lysosomal escape, Tumor homing-penetrating peptide, Chemotherapy enhancement, PD-L1 blockade

## Abstract

**Background:**

Recent studies have demonstrated that multidrug resistance (MDR) is a critical factor in the low efficacy of cancer chemotherapy. The main mechanism of MDR arises from the overexpression of P-glycoprotein (P-gp), which actively enhances drug efflux and limits the effectiveness of chemotherapeutic agents.

**Results:**

In this study, we fabricated a “combo” nanoagent equipping with triple synergistic strategies for enhancing antitumor efficacy against MDR cells. Tumor homing-penetrating peptide endows the nanosystem with targeting and penetrating capabilities in the first stage of tumor internalization. The abundant amine groups of polyethylenimine (PEI)-modified nanoparticles then trigger a proton sponge effect to promote endo/lysosomal escape, which enhances the intracellular accumulation and retention of anticancer drugs. Furthermore, copper tetrakis(4-carboxyphenyl)porphyrin (CuTCPP) encapsulated in the nanosystem, effectively scavenges endogenous glutathione (GSH) to reduce the detoxification mediated by GSH and sensitize the cancer cells to drugs, while simultaneously serving as a photoacoustic imaging (PAI) contrast agent for image visualization. Moreover, we also verify that these versatile nanoparticles in combination with PD-1/PD-L1 blockade therapy can not only activate immunological responses but also inhibit P-gp expression to obliterate primary and metastatic tumors.

**Conclusion:**

This work shows a significant enhancement in therapeutic efficacy against MDR cells and syngeneic tumors by using multiple MDR reversing strategies compared to an equivalent dose of free paclitaxel.

**Graphic Abstract:**

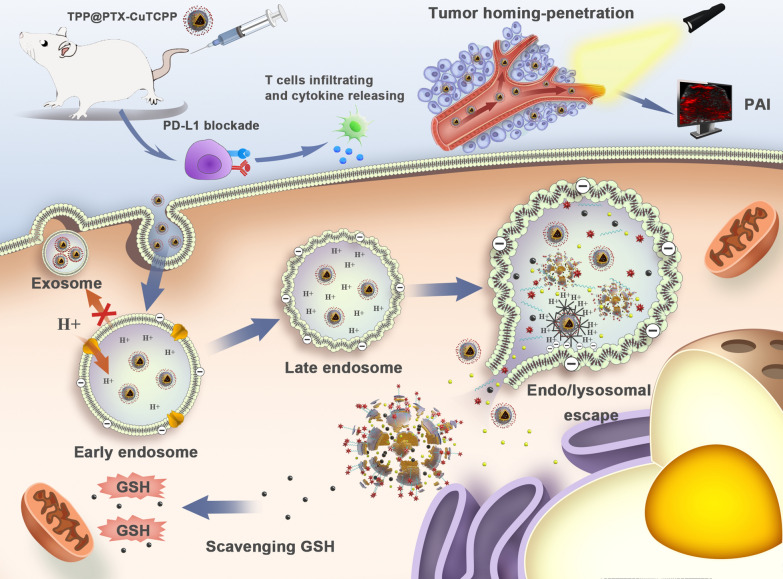

**Supplementary Information:**

The online version contains supplementary material available at 10.1186/s12951-021-00947-9.

## Introduction

For decades, cancer has been one of the greatest contributors to the high mortality worldwide, therefore, considerable attention has been given to developing effective anticancer methods, such as photothermal therapy [1], magnetothermal therapy [2], photodynamic therapy and chemotherapy [3, 4]. Although chemotherapy remains an effective approach to inhibiting drug-sensitive cancer cells, a common major obstacle that emerges when using chemotherapeutic agents is intrinsic or acquired multidrug resistance (MDR) [5, 6]. The main mechanism of MDR involves the overexpression of P-glycoprotein (P-gp) or other multidrug resistance protein (MRP)-associated efflux pumps. Under selection pressure, these efflux pumps enhance drug efflux via membrane transporters, export antitumor drugs and prevent effective cellular uptake [7, 8]. Reducing the intracellular retention and concentration of drugs in cancer cells with MDR makes chemotherapy inefficient and fails to achieve the clinical demand. Although MDR inhibitors are one of most commonly used strategies to overcome MDR, nonspecific agents might downregulate P-gp expression in the cells of normal tissues that play a physiological role in excreting xenobiotics, probably leading to the destruction of homeostasis [9]. Currently, various nanoscale agents, such as, noble metals (Ag, Au) [10, 11], mesoporous silica [12], liposomes [13], polymers [14] and their hybrids, have been systematically investigated and have drawn increasing attention because they offer several favorable properties in the fields of drug delivery, tissue engineering, imaging sensors, etc. [15, 16]. However, these nanoparticles (NPs) with particle sizes ranging from 50 to 500 nm are mostly internalized via endocytosis and trapped in recycling endosomes (pH 5.5–6.5) [17, 18]. Subsequently, some of the endosomes are either transported out of cells by fusion with the plasma membrane in the form of exosomes or integrated with lysosomes, whose contents can be quickly deactivated in the latter case [19, 20]. Therefore, reduced accessibility of the hydrophobic chemotherapeutic agents to their target sites is found due to endo/lysosomal sequestration, resulting in marked drug resistance. Another chemotherapeutic challenge in MDR cells is a mass of cytosolic antioxidants, typically glutathione (GSH) [3, 21, 22]. As the most abundant nonprotein thiol found in all cellular compartments, GSH presents mostly in the cytoplasm (85%) in its reduced form and is a major cellular redox component in living organisms [23, 24]. It has been demonstrated that endogenous GSH has an upregulated concentration (up to 10 mM) in many drug-resistant tumor cells, which is notably higher than that in normal cells (approximately two- to fourfold higher) [25]. Through reducing or binding to chemotherapeutic agents such as paclitaxel (PTX), doxorubicin (DOX), or cisplatin, GSH-mediated detoxification plays a critical role in protecting important cellular components, preventing damage to DNA or proteins and inducing the loss of drug efficacy [26–28].

Numerous studies concentrating on the reversal of MDR have shown moderate progress [29–31], however, these notable results are restricted by their use of a single reversal strategy that acts on only one mechanism of drug resistance. Thus, multiple synergistic strategies are urgently required to overcome various resistance mechanisms, such as the low intracellular retention of drugs and abundant GSH in MDR cells. Polyethylenimine (PEI) is generally recognized as an effective material that can facilitate endo/lysosomal escape due to its high amine density and buffering capacity. Compared to commonly used PEI (Mw: 25,000 Da), low molecular weight PEI (Mw: 1800 Da) has been suggested to possess better biocompatibility and degradability [32, 33]. Under the weakly acidic conditions of tumor cells, the abundant amine groups in PEI can be protonated, which triggers the large inflow of protons, chloride counterions, and water into the endo/lysosomes, promoting endo/lysosomal rupture through high osmotic pressure which is known as the “proton-sponge effect” [34]. This trait helps to enhance the intracellular retention of drugs loaded by nanocarriers after endocytosis and avoid the enzymatic degradation of drugs by lysosomes [35]. In addition, a GSH consumption strategy was simultaneously performed in present study. An increasing amount of evidence has suggested that CuTCPP, consisting of Cu^2+^ and tetrakis(4-carboxyphenyl)porphyrin (TCPP) ligands, has a distinct GSH scavenging effect through the cycle conversion of Cu^2+^ into Cu^1+^ in CuTCPP [36–38]. Thus, CuTCPP was encapsulated into the core of poly(lactic acid-*co*-glycol acid) (PLGA)-based NPs to sensitize cancer cells to chemotherapy, the so-called chemosensitization, via Cu^2+^-induced GSH depletion along with a pro-oxidant [39]. In addition, because of its good optical absorption properties in the near infrared (NIR) region, CuTCPP is considered as an excellent candidate for photoacoustic imaging (PAI), a promising imaging mode due to its noninvasiveness and high spatial resolution. It is therefore believed that a nanoplatform containing CuTCPP can serve as a favorable PAI enhanced-contrast agent for image visualization and monitoring during the process of treatment [40]. We have previously reported a series of tumor homing-penetrating peptide (THPP)-functionalized NPs that specifically recognize the endothelium of tumor vessels and transport the attached cargo into the cells [41, 42]. As a seven amino acid truncated form peptide of LyP1(CGNKRTRGC), tLyP-1 (CGNKRTR) has been demonstrated to be a ligand that targets to the neuropilin-1 (NRP-1) receptor, which is overexpressed in breast cancer and several human tumor types. Moreover, by actively transferring through tumor tissue barriers, such as the extracellular matrix barrier and vascular endothelial barrier, the emerging peptide readily promotes NPs to permeate deeper into solid tumor regions that lack microvessels for further enhancement of reversal efficiency [43, 44]. In this case, we expect this powerful THPP to endow the nanosystem with tumor targeting and penetrating abilities in the first stage of tumor internalization to assist in overcoming MDR.

Notably, the use of different types of cancer immunotherapies including cancer vaccines, cytokine therapy, adoptive T-cell transfer and checkpoint blockade, have achieved exciting outcomes in the clinic [45, 46]. Some recent studies revealed that PD-1/PD-L1 inhibition may downregulate PI3K/AKT and MAPK/ERK pathway-mediated MDR1/P-gp expression in breast cancer cells, suggesting that this effect probably contributes to increased chemotherapy efficacy in breast cancer patients [47–49]. On the other hand, it has been reported that secretion of pro-inflammatory cytokines or chemokines such as tumor necrosis factor (TNF), alpha-interferon (IFN) and interleukin-6 (IL-6) triggered by the immune system is also related to expression of various MRP types. Although immune therapy for overcoming MDR tumors is not an optimal approach at this early juncture, immune-chemotherapy as a promising strategy combining cytotoxic agents with immune strategy for combating MDR tumors appears to be feasible in theory [50].

**Scheme 1.**
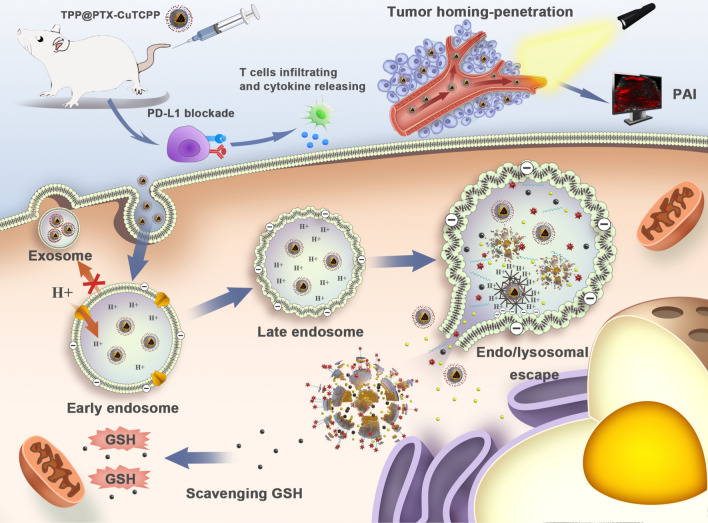
Schematic illustration on reversal of multidrug resistance by triple synergistic strategies together with PD-L1 blockade therapy, involving endo/lysosomal escape, GSH depletion and tumor-homing penetrating capabilities, which are equipped in one versatile nanoparticle

Herein, we synthesized the versatile NPs namely, tLyP-1-PEI-PLGA-PTX-CuTCPP (TPP@PTX-CuTCPP), which are equipped with triple MDR reversal strategies for tumor chemotherapy amplification, as illustrated in Scheme 1. The tLyP-1-PEI functionalized PLGA shell endowed the multitasking nanoagent with endo/lysosomal escape and tumor homing-penetrating behavior providing the NPs with outstanding intracellular drug retention and allowing their rapid crossing of the tumor endothelial barrier. Depleting high-level GSH followed after the codelivery of CuTCPP to recover PTX in MCF-7/Taxol cells. Moreover, a strategy in which multifunctional NPs are further combined with PD-L1 checkpoint blockade, which is expected to suppress P-gp expression and activate cancer immunotherapy, was developed. The anticancer efficacy and imaging ability of these NPs were evaluated in vitro and in vivo.

## Experimental

### Materials and reagents

Poly(lactic-*co*-glycolic acid) (PLGA, lactide:glycolide = 50:50, molecular weight [MW] 15,000 Da) was purchased from Jinan Daigang Biomaterial Co., Ltd. (Shandong, China). poly(vinyalcohol) (PVA, MW 25,000 Da) and polyethylenimine (PEI, MW 1800 Da) were purchased from Sigma-Aldrich (St Louis, MO, USA). Paclitaxel (PTX) was purchased from Aladdin Bio-Chem Technology Co., Ltd (Shanghai, China). Cu-tetrakis(4-carboxyphenyl)porphyrin and FITC-labeled PTX were purchased from Ruixi Biotech Co., Ltd (Xi’an, China). tLyP-1 (CGNKRTR) was synthesized by Chinapeptide (Shanghai, China). MCF-7, MCF-7/Taxol and HUVECs were purchased from the MEIXUAN Biological science and technology Co., Ltd (Shanghai, China). GSH assay kit was purchased from Nanjing Jiancheng Bioengineering Institute (Nanjing, China). LysoTracker Green DND-26 and agarose were purchased from Invitrogen (Thermo Fisher Scientific). Cell Counting Kit-8 (CCK-8) Calcein-AM, and PI were obtained from Dojindo (Japan). P-gp Rabbit pAb was obtained from ABclonal (Wuhan, China). Anti-PD-L1 was purchased from BioXcell (USA). Tumor necrosis factor alpha, interferon gamma and interleukin 6 ELISA kit were purchased from Uscn Life Science, Inc., (China). Anti-CD3^+^-FITC, anti-CD8^+^a-APC and anti-CD4^+^-PC5.5 were purchased from Biolegend (USA). Deionized water obtained from the Millipore system (Direct-Q 5, FRA) was used in all preparations.

### Synthesis of tLyP-1-PEI

20 mg tLyP-1 was first dissolved completely in 5 mL of methanol, followed by the addition of 6—(maleic imide) caproic acid succinimide ester (40.69 mg, 0.132 mmol) at room temperature for 30 min. Subsequently, PEI (237.6 mg, 0.132 mol) and triethylamine (0.072 mol L^−1^, 5 mL) were added into the reaction at room temperature for another 1 h. Finally, organic solvents were removed by vacuum-rotary evaporation procedure and the intermediate solution was dialysed (MW, cut-off = 3500 Da) against distilled water for 48 h to remove untreated stuff and lyophilization. The product was denoted as tLyP-1-PEI.

### Synthesis of TPP@PTX-CuTCPP

The nanoparticles encapsulating PTX and CuTCPP were synthesized by using a modified double emulsion strategy. Initially, PTX (5 mg) was added into PLGA (50 mg) dissolved in trichloromethane (2 mL) as the stock solution and CuTCPP aqueous solution (400 μL, 12.5 mg mL^−1^) was then put in the above solution. Afterwards, the mixture was firstly emulsified through ultrasonic probe (Sonics & Materials Inc., USA) with power of 75 W for 3 min. For the second emulsion, PVA solution (5.5 mL, w/v = 4%) was poured into above solution for homogenization at 45 W for 2 min. Isopropyl alcohol solution (12.5 mL, v/v = 2%) was then poured in final emulsion to evaporate organic solvent for 3–4 h at room temperature. Finally, the PLGA-PTX-CuTCPP (P@PTX-CuTCPP) was obtained after centrifugation at 12,000 rpm for 5 min. The preparation of PLGA-PTX (P@PTX) was performed similar to the above process except that CuTCPP solution was replaced by deionized water at same volume. The similar method also was applied to the synthesis of PLGA-CuTCPP (P@CuTCPP) except removing PTX from stock solution. The P@PTX-CuTCPP was further coupled to tLyP-1-PEI. Briefly, the carboxylic groups on the surfaces of PLGA was activated by adding into 2-(*N*-morpholino) ethanesulfonic acid (MES) buffer solution (0.05 mM, pH 5.5) containing with of *N*-hydroxysuccinimide (NHS) and *N*-(3-dimethylaminopropyl)-*N*ʹ-ethylcarbodiimide hydrochloride (EDC) and the mixture was incubated in a shaker for 2 h at room temperature to activate the terminal carboxyl groups. Secondly, MES buffer solution (0.05 mM, pH 8.0) was used to dissolve the mixture again after centrifugation. Thirdly, tLyP-1-PEI (10 mg) was added and allowed to react for another 12 h. Finally, the resulting tLyP-1-PEI-PLGA-PTX-CuTCPP (TPP@PTX-CuTCPP) was purified by repeated washing 3 times with deionized water to remove unreacted materials. The preparation PEI-PLGA-PTX-CuTCPP (PP@PTX-CuTCPP) was prepared similar to the above method excepting that tLyP-1-PEI was replaced by PEI (10 mg).

### Characterization of TPP@PTX-CuTCPP

The morphology of TPP@PTX-CuTCPP was observed by scanning electron microscopy (SEM, Hitachi S-3400N, Japan) and transmission electron microscopy (TEM, Hitachi H-7600, Japan). The zeta potentials and size distributions were determined by a dynamic light scattering (DLS, Malvern, NanoZS, UK). The absorption spectrum of TPP@PTX-CuTCPP, tLy-1-PEI-PLGA-PTX-CuTCPP (TPP@PTX), and CuTCPP aqueous solutions were measured by UV–vis spectroscopy (Shimadzu, UV-3600, Japan) to observe the presence of CuTCPP in the NPs. The synthesis of tLyP-1-PEI and tLyP-1-PEI-PLGA were detected by nuclear magnetic resonance spectroscopy (NMR, AVANCE III, Bruker, Germany), high performance liquid chromatography (HPLC, Waters, ZQ2000, China), Fourier transform infrared spectroscopy (FTIR, ThermoFisher, iS50, American) and time of flight mass spectrometer (TOF–MS, BAIIENS, AB5800, China). The standard curve of free CuTCPP was calculated to evaluate the amount of CuTCPP encapsulated into PLGA. The entrapment efficiency and content of CuTCPP were calculated by Eq. (1).

1 $$ {\text{CuTCPP~entrapment~efficiency}}\,(\% ) = \frac{{\text{Mass~of~total~CuTCPP}} - ~{\text{Mass~of~unentrapped~CuTCPP}}}{{{\text{Mass~of~total~CuTCPP}}}}\times 100\%  $$

The spectrum of PTX solutions at different concentrations, and TPP@PTX-CuTCPP (dissolved in DMSO and methyl alcohol (v/v) = 1:1) were measured by HPLC system to calculate standard concentration curve for analyzing the amount of PTX in TPP@PTX-CuTCPP. The entrapment efficiency and content of PTX were calculated by Eq. (2).

2 $$ {\text{PTX~entrapment~efficiency }}(\% ) =\frac{{{\text{Mass~of~entrapped~PTX}}}}{{{\text{Mass~of~total~PTX}}}}\times 100\%$$

### Drug release

Firstly, TPP@PTX-CuTCPP suspension was equivalently injected into two dialysis bags (n = 3, MW cutoff 8000–14,000 Da). The dialysis bags were placed in a plastic bottle containing 60 mL buffer solution containing with ethanol (v/v = 30%), sodium azide (w/v = 0.02%) and tween-80 (v/v = 0.1%) at different pH values (pH = 6.3 and 7.4) under 150 rpm stirring at room temperature. At every predetermined time point (preinjection and 1, 3, 7, 12, 18, and 24 h), 1 mL of buffer solution in the plastic bottle were collected for measurement by DLS and HPLC system and replaced by 1 mL of fresh buffer solution into the plastic bottle at the same time. Finally, variation of size distributions and cumulative release ratio in two groups were calculated.

### Cell culture and tumor-bearing animal model establishment

MCF-7/Taxol cells were maintained in RPMI-1640 medium (HyClone, USA) supplemented with 20% fetal bovine serum (FBS, Bioind, Israel), 100 U mL^−1^ penicillin and-streptomycin and 0.5 μg mL^−1^ PTX solution. MCF-7 cells were maintained in 1640 medium supplemented with 10% FBS and 100 U mL^−1^ penicillinand-streptomycin solution. HUVECs were maintained in RPMI-1640 medium supplemented with 10% FBS and 100 U mL^−1^ penicillin and-streptomycin solution. The cells were maintained in an incubator containing 5% CO_2_ atmosphere at 37 ℃. All female BALB/c nude mice (16 to 20 g, 4 to 6 weeks) were bred in humidity conditions between 18 and 20 ℃. Food and water were free to provide. All the animal experiments were approved by Institutional Animal Care and Use Committee of Chongqing Medical University and all procedures were in accordance with the ethical standards Use Committee of Chongqing Medical University. The clearance of all laboratory animal carcasses followed animal ethical clearance principle (SYXK2018-0003). To establish the MCF-7/Taxol tumor models, 4 × 10^6^ MCF-7/Taxol cells suspended in PBS solution (100 μL) were subcutaneously injected into the right flank of each nude mouse. To establish an artificial bilateral tumor model, 2 × 10^6^ 4T1 cells suspended in PBS solution (100 μL) were orthotopically inoculated into the right breast fat pad of the 30 BALB/c mice as primary tumor. 7 days later, 4T1 cells were injected into the left breast fat pad as distant tumor. The tumor-bearing mice were used for additional in vivo experiments and the volume of tumors were calculated by the following equation

$$ \left[ {\pi /6 \times {\text{length}} \times \left( {{\text{width}}} \right)^{2} } \right]. $$

### Cell targeting and tumor spheroid penetrating efficiency

MCF-7/Taxol and Human umbilical vein endothelia cells (HUVECs) were seeded into a laser confocal cell-culture dish at a density of 2 × 10^5^ cells per well respectively. After 24 h incubation, the cell culture medium was replaced with the medium containing DiI-labeled (λ_excitation_/λ_emission_ = 549 nm/565 nm) TPP@PTX-CuTCPP or PP@PTX-CuTCPP for 0.5, 1, and 2 h, respectively. For the qualitative study, the cells were washed three times with PBS and fixed with 4% paraformaldehyde for 15 min at room temperature. The cell nuclei were then stained with DAPI for 10 min. Finally, the fluorescence images of the treated cells were acquired by confocal laser scanning microscopy (CLSM, Nikon, A1, Japan). In addition, the quantitative intracellular uptake of TPP@PTX-CuTCPP or PP@PTX-CuTCPP for MCF-7/Taxol and HUVEC cells at different intervals were analyzed by flow cytometry (Becton Dickinson, FACS Vantage, USA). Three dimensional spheroids of MCF-7/Taxol cells were established by seeding cells in ultra-low adherent plates. Briefly, 1 × 10^5^ MCF-7/Taxol cells per well were seeded in 6-well ultra-low adherent plates for 10 days formation. The spheroids were then coincubated with DiI-labeled TPP@PTX-CuTCPP or PP@PTX-CuTCPP for 6 h and send for CLSM observation after washing twice with PBS, respectively. Multiple level scans for three dimensional tumor spheroids were done at 2 μm intervals to measure penetration depth.

### In vitro and in vivo PAI

To determine the photoacoustic imaging (PAI) performance of TPP@PTX-CuTCPP, a 3% agarose gel phantom with a hole was initially constructed. The PAI images were obtained by a photoacoustic imaging system (Vevo LAZR, Canada). TPP@PTX-CuTCPP (200 μL, 250 μg mL^−1^) was used for full wavelengths scanning to detect the maximum absorbance. Varied CuTCPP concentrations of TPP@PTX-CuTCPP (125, 150, 175, 200, 225, 250 μg mL^−1^) dissolved in deionized water were added to acquire the corresponding photoacoustic images by Vevo LAZR software at the excitation in 690 nm. The quantified photoacoustic value within tumor region of each image was then analyzed. For in vivo PAI, MCF-7/Taxol tumor-bearing mice were intravenously injected with TPP@PTX-CuTCPP saline solution. Then, the PAI images were obtained at various time intervals (preinjection and, 1, 2, 6, 12 and 24 h postinjection) and the photoacoustic intensity in the tumor regions was measured.

### Intraellular uptake and endosomal escape

The cellular uptake of DiI-labeled PP@PTX, P@PTX, and free FTIC-PTX (λ_excitation_/λ_emission_ = 488 nm/525 nm) was visualized by CLSM. MCF-7/Taxol cells were seeded into confocal dishes at a density of 2 × 10^5^ cells per well. After 24 h incubation, the cell culture medium was replaced with the serum-free medium containing DiI-labeled PEI-PLGA-PTX (PP@PTX), P@PTX or free FITC-PTX for 6 h of incubation. The cells were washed with PBS three times after incubation and treated with serum-free cell culture medium for another 2 h, 4 h, 6 h, 8 h, 10 h, 12 h respectively. At predetermined time point, cells were rinsed to remove effluent PTX and then lysed with RAPI cell lysis buffer (Beyotime, China). The fluorescence intensities of intracellular PTX (n = 3) were calculated by using a microplate reader (Bio-Tek Instrument Inc. USA) and observed by CLSM. To visualize the endo/lysosomal escape properties of NPs, the endo/lysosomes were stained to observe the colocalization of intracellular PP@PTX and P@PTX. After 6 h coincubation with the medium containing DiI-labeled PP@PTX and P@PTX, the culture medium was replaced with fresh medium containing Lysotracker (λ_excitation_/λ_emission_ = 504 nm/511 nm) and incubated for another 0.5 h to stain the endo/lysosomes. The cells were gently washed with PBS and stained with DAPI for 10 min. Finally, the fluorescence of NPs and cells were visualized with the CLSM. The co-localization of NPs and Lysotracker from five representative cell images was quantified with Fuji ImageJ software by calculating the Pearson’s correlation coefficient. To investigate the endo/lysosomal membrane integrity, calcein was added in the presence of PP@PTX or P@PTX and coincubated with cells for 6 h. After three PBS washing steps, the cells were captured by CLSM.

### GSH scavenging effects in solution and cells

The aliquots were prepared by mixing GSH (200 μL, 1 mM) with free CuTCPP at varied concentration (12.5, 25, 50, 100, 200 μg mL^−1^), P@CuTCPP (containing CuTCPP at same concentration), or deionized water (n = 3), respectively. The supernatant was then extracted from respective aliquot at predetermined time point (24 h) for GSH assay kit and X-ray photoelectron spectroscopy (XPS, ThermoFisher, ESCALAB250, USA) according to the manufacturer’s protocol. For detection of GSH in cells, firstly, MCF-7/Taxol cells were seeded in 6-well culture plates at a density of 2 × 10^5^ cells. After 24 h incubation, cell culture medium were replaced with NP-free medium, medium containing PLGA with free BSO (45 μg mL^−1^), or P@CuTCPP containing concentration of CuTCPP (30 μg mL^−1^) for another 24 h incubation. The cells were centrifuged at 1000 rpm for 5 min after counting and normalizing the numbers of cells in each group and disrupted by ultrasonic wave (200 w, 5 min). The samples were centrifuged at 10,000 rpm for 10 min at 4 °C to collect supernatant that was used for GSH assay.

### Cell viability and cellular apoptosis assay

The MCF-7/Taxol and HUVEC cells were initially seeded in 96-well culture plates for 24 h. Then, the culture medium was replaced with the medium containing TPP@CuTCPP at different concentrations based on PLGA (100 μL, 0.3125, 0.675, 1.25, 2.5 and 5 mg mL^−1^). After 24 h of treatment, the CCK-8 assay was used to evaluate the viability of cells according to the manufacturer’s protocol. The optical density (OD) at 450 nm was read with a microplate reader. To assess the anticancer efficacy in vitro, the MCF-7/Taxol cells were treated by the following eight groups (n = 3): G1, control; G2, free PTX; G3, P@PTX; G4, P@PTX-CuTCPP; G5, PP@PTX; G6, TPP@PTX; G7, PP@PTX-CuTCPP; and G8, TPP@PTX-CuTCPP. Firstly, MCF-7/Taxol cells were seeded in 96 well plates at a density of 1 × 10^4^ cells per well. 24 h later, fresh medium containing different NPs and free drugs with equivalent concentration of PTX (50 μg mL^−1^) were added to replace the previous medium and coincubated with the cells for another 24 h. Finally, the cell viabilities were evaluated by CCK-8 assay. We performed cellular apoptosis assay by treating MCF-7/Taxol cells with the same above condition after incubation in 6-well culture plates for 24 h. The cells were then collected in 200 μL of binding buffer. PI and Annexin V-FITC were added for coincubation with the cells for 15 min. The stained cells were analyzed using a flow cytometry.

### Living and dead cell co-staining experiment

MCF-7/Taxol cells were seeded into a laser confocal cell-culture dish at a density of 2 × 10^5^ cells per well for 24 h incubation, the cells were treated by the following eight groups and incubated for another 24 h: G1, control; G2, free PTX; G3, P@PTX; G4, P@PTX-CuTCPP; G5, PP@PTX; G6, TPP@PTX; G7, PP@PTX-CuTCPP; and G8, TPP@PTX-CuTCPP with concentration of 50 μg mL^−1^ based on PTX. The therapy effects were detected by inverted fluorescence microscope (FL, Olympus IX53, Canada) after costaining with 40 nM calcein AM and 4.5 μM propidium iodide (PI) in PBS buffer solution for 10 min at 37 °C. PI was excited at 633 nm and detected with a 660–710 nm bandpass filter. Calcein AM was excited at 488 nm and detected with a 500–550 nm bandpass filter.

### Anticancer effects in vivo

Forty tumor-bearing nude mice were randomly divided into eight groups (n = 5): G1, Control; G2, Free PTX; G3, P@PTX; G4, P@PTX-CuTCPP; G5, PP@PTX; G6, TPP@PTX; G7, PP@PTX-CuTCPP; and G8, TPP@PTX-CuTCPP. Corresponding formulations were injected into MCF-7/Taxol tumor-bearing mice intravenously at the equivalent dose of 2 mg PTX kg^−1^ per mouse 4 times every 4 days. The control group was injected with saline solution (200 μL). The tumor-volume changes of each group were recorded and the weight of mice in different groups was monitored every 4 days. Tumor volume changes were normalized using the relative tumor volumes (initial tumor volume (V_0_)/current tumor volume (V)). On day 16, all mice were sacrificed for tumor dissection. Targeted tumor tissue was stained with Proliferating cell nuclear antigen (PCNA), TdT-mediated dUTP nick-end labeling (TUNEL), and hematoxylin–eosin (H&E) for immunohistochemical analysis. The main organs of the mice were excised and fixed for H&E staining.

### Anticancer efficacy of combined chemotherapy and immunotherapy

The bilateral tumor-bearing mice were then randomly divided into six groups (n = 5): (1) control; (2) control + PD-L1; (3) free PTX; (4) free PTX + PD-L1; (5) TPP@PTX-CuTCPP and (6) TPP@PTX-CuTCPP + PD-L1. The day was defined as day 0 when treatments were performed. Corresponding formulations were intratumorally injected into primary tumors (first tumors) of 4T1 tumor-bearing mice at the equivalent dose of 1 mg PTX kg^−1^ per mouse 3 times every 6 days. The control group was injected with saline solution (200 μL). Furthermore, anti-PD-L1 (1.5 mg kg^−1^ per mouse) were intravenously injected to the mice in control + PD-L1 group, free PTX + PD-L1 group and TPP@PTX-CuTCPP + PD-L1 group on day 1, 4, 7 and 11. To evaluate the antitumor efficacy of combined chemotherapy and anti-PD-L1, the tumor-volume changes of each group were recorded. Tumor volume changes on both sides were normalized using the relative tumor volumes (initial tumor volume (V_0_)/current tumor volume (V)). On day 16, all mice were sacrificed for tumor dissection hematoxylin–eosin (H&E) for immunohistochemical analysis. Western blot was used to investigate the expression of P-gp in tumor cells. To assess the in vivo antitumor immune responses against distant tumors, the tumors were harvested and treated with 0.2% collagenase D, 0.01% hyaluronidase and 0.002% DNase to produce a single-cell suspension. Flow cytometry was used to determine the proportions of tumor-infiltrating T cells (CD3^+^, CD4^+^ and CD8^+^ T cells). TNF-α, IFN-γ, IL-6, IL-12 were examined by using ELISA kits according to the manufacturer's protocols. CD3^+^, CD4^+^, and CD8^+^ T cells in the distant tumor tissue section were stained for immunofluorescence.

### Fluorescence imaging and biodistribution in vivo

For the fluorescence imaging (FL) and biodistribution assessment in vivo, MCF-7/Taxol tumor-bearing mice (n = 3) were injected with DIR-labiled (λ_excitation_/λ_emission_ = 748 nm/780 nm) TPP@PTX-CuTCPP or PP@PTX-CuTCPP solution via tail vein. FL images were acquired at preinjection and, 2, 6, and 24 h postinjection by using fluorescence imaging system (CRi Inc, USA), and the relative fluorescence intensity of the tumor regions was recorded. The tumor tissues and major organs were harvested for ex vivo experiment through FL.

### Biosafety assay

Twenty-four BALB/c nude mice were randomly divided into five groups (n = 4). Twenty mice were sacrificed at predetermined time points (1, 3, 7, 14, and 28 days) after postinjection of 200 μL TPP@PTX-CuTCPP at a dose of 5 mg mL^−1^ based on PLGA and four mice in control group after postinjection fo saline solution were sacrificed at 1 day. The blood samples were further collected for routine blood examination and biochemistry assay including l-lactate dehydrogenase, creatine kinase, alanine aminotransferase (ALT), aspartate transaminase (AST), total bilirubin, creatinine (CR), and urea nitrogen (BUN). The major organs of the mice were harvested and fixed with 4% polyoxymethylene and stained with H&E for histological analysis.

### Statistical analysis

All statistical analyses were analyzed by SPSS software 20.0 (Chicago, USA). Quantitative data were presented as the mean ± standard deviation (SD). The significance of the data was analyzed according to the one-way ANOVA tests and Student’s t-test. The level of significance in the statistical analyses was defined as *p < 0.05, **p < 0.01.

## Results and discussion

### Design, synthesis, and characterization of TPP@PTX-CuTCPP

**Fig. 1:**
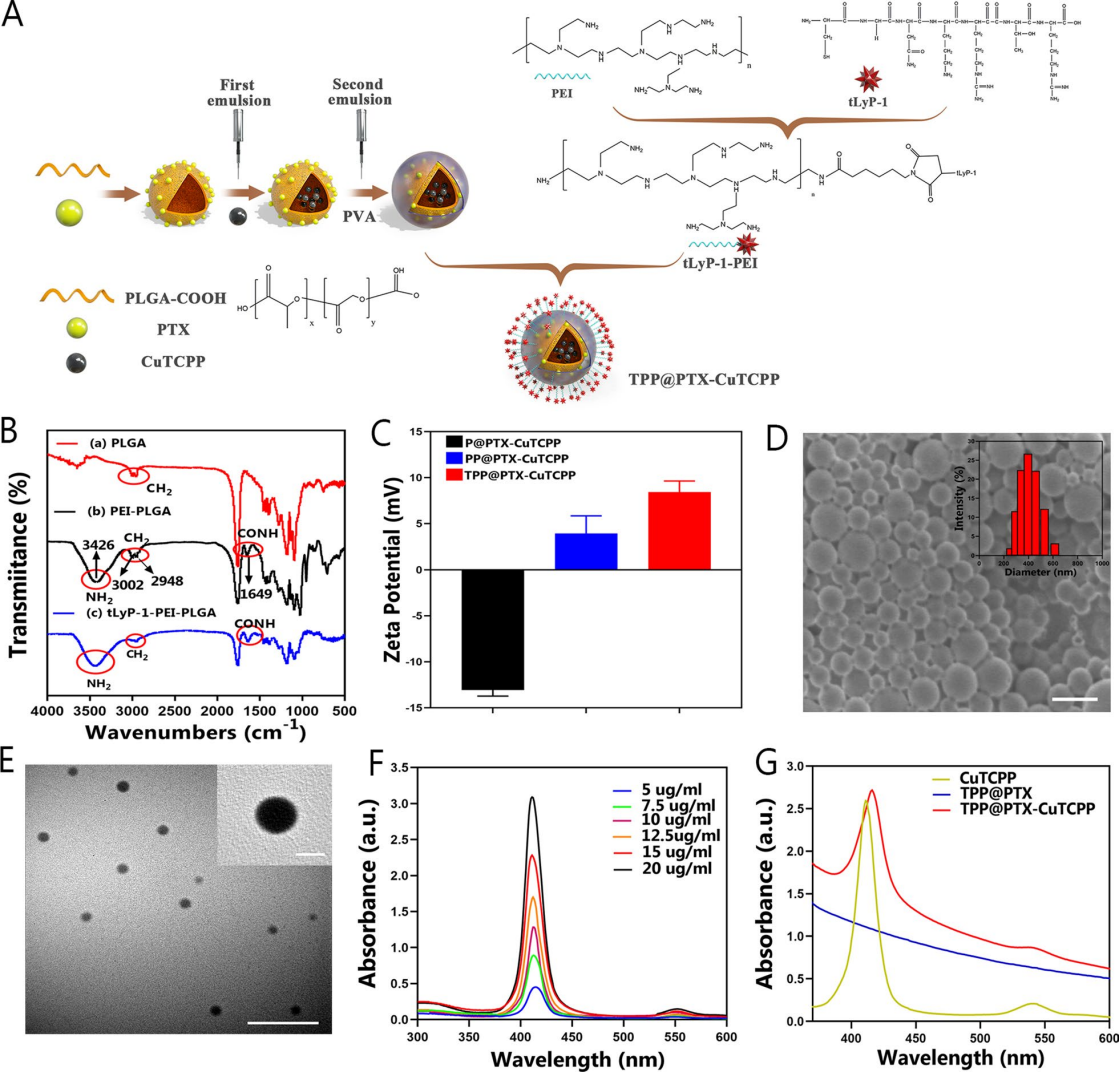
Synthesis scheme and characterizations of TPP@PTX-CuTCPP nanoparticle. **A** Schematic on the synthetic procedure of TPP@PTX-CuTCPP. **B** FTIR spectrum of different samples including PLGA (a) PEI-PLGA (b), and tLyP-1-PEI-PLGA (c). Based on curve a, curve b and c have characteristic peaks around 3426 and 1649 cm^−1^ attributed to the stretching vibrations of the amide bond. **C** Zeta ptentials of P@PTX-CuTCPP, PP@PTX-CuTCPP, and TPP@PTX-CuTCPP. **D** An SEM image of TPP@PTX-CuTCPP, the scale bar is 0.5 μm (inset: size distribution based on diameter, intensity (%)). **E** A TEM image of TPP@PTX-CuTCPP NPs, the scale bar is 1 μm (inset: scale bar is 200 nm). **F** UV–vis absorbance spectra of CuTCPP at elevated concentrations. **G** UV–vis absorbance of free CuTCPP, TPP@PTX, and TPP@PTX-CuTCPP

The synthesis process of TPP@PTX-CuTCPP NPs was accomplished in two steps, nanoplatform preparation and surface modification, as depicted in Additional file 1: Scheme S1 and Fig. 1A. In this work, PLGA acted as the nanocarrier for the coencapsulation of a hydrophilic molecule, i.e., CuTCPP, and a hydrophobic molecule i.e., PTX, via a typical double-emulsion process. We then confirmed the sound synthesis of tLyP-1 by mass spectrometry (Thermo Fisher, TSQ Altis, USA), which demonstrated a comparable molecular weight (833.97 Da) to that of the short peptide (CGNKRTR) (Additional file 1: Figure S1). Next, tLyP-1-PEI was prepared using the maleimide-thiol method as determined by NMR. The NMR spectrum displayed peaks from 2.6 to 3.0 ppm compared with the spectrum of PEI (peaks from 2.5 to 2.9 ppm), which were attributed to the CH_2_-N stretch of PEI, indicating the successful conjugation of tLyP-1 with PEI. In addition, the tumor-targeting feature was preserved by the modification process because the C-terminal sequence of tLyP-1 was not altered (Additional file 1: Figure S2). The disappearance of characteristic peaks of tLyP-1 from HPLC also validated the synthesis of tLyP-1-PEI (Additional file 1: Figure S3). Finally, tLyP-1-PEI was covalently bonded onto the surface of the nanosystem via a carbodiimide coupling reaction as characterized by FTIR. The peaks at 3002 cm^−1^ and 2948 cm^−1^ in the tLyP-1-PEI-PLGA and PEI-PLGA spectra can be attributed to the C–H stretch of PLGA. Evident characteristic peaks at 1649 cm^−1^ and 3426 cm^−1^ associated with the –CO–NH and –NH_2_ groups in tLyP-1-PEI-PLGA and PEI-PLGA provided direct evidence of the existence of amide bonds (Fig. 1B) [51, 52]. The zeta potentials of the NPs modified with tLyP-1-PEI or only PEI were increased to a positive value attributable to the positive charge from either the tLyP-1 peptide or PEI compared with the pristine NPs, according to DLS measurements (Fig. 1C). Moreover, the molecular weight of nanosystem increased from 14,955.22 to 20,722.68 Da after tLyP-1-PEI modification, as detected by TOF–MS. This may be explained by more than one tLyP-1-PEI monomers tethering to PLGA, which also suggested again the successful binding of tLyP-1-PEI to NPs (Additional file 1: Figure S4). The morphology of the obtained TPP@PTX-CuTCPP NPs was then revealed by SEM and TEM. These NPs displayed well-defined appearance and monodispersed distribution with a size of approximately 300 nm size, which was comparable to 354.8 nm as measured by DLS (Fig. 1D, E). In addition, the TPP@PTX-CuTCPP NPs had a polydispersity index (PDI) of 0.14 in deionized water, indicating their high homogeneity, which matched well with the results of fluorescence and optical imaging (Additional file 1: Figure S5). In particular, TPP@PTX-CuTCPP exhibited good stability in various physiological solutions such as water, PBS, and RPMI-1640 culture medium containing FBS (Additional file 1: Figure S6). Subsequently, UV–vis spectroscopy and HPLC were performed to confirm the successful loading and calculate the entrapment efficiency of the cargoes via their concentration and related absorbance (Fig. 1F). The amount of CuTCPP loaded in TPP@PTX-CuTCPP was quantified by UV–vis spectroscopy, which demonstrated a characteristic absorption peak at a wavelength of 415 nm, which was slightly redshifted from 412 nm. However, no characteristic absorption peak was observed in NPs without encapsulation of CuTCPP (Fig. 1G). Digital photographs also validated the successful encapsulation of CuTCPP due to the appearance of the aqueous solution changing from white to pink after CuTCPP loading (Additional file 1: Figure S7). The CuTCPP entrapment efficiency was determined to be 27.1% by UV–vis spectroscopy. Additionally, the entrapment efficiency of PTX was approximately 70.1%, with a characteristic band at 227 nm by HPLC (Additional file 1: Figures S8, S9).

### Intracellular uptake and endo/lysosomal escape efficiency

**Fig. 2:**
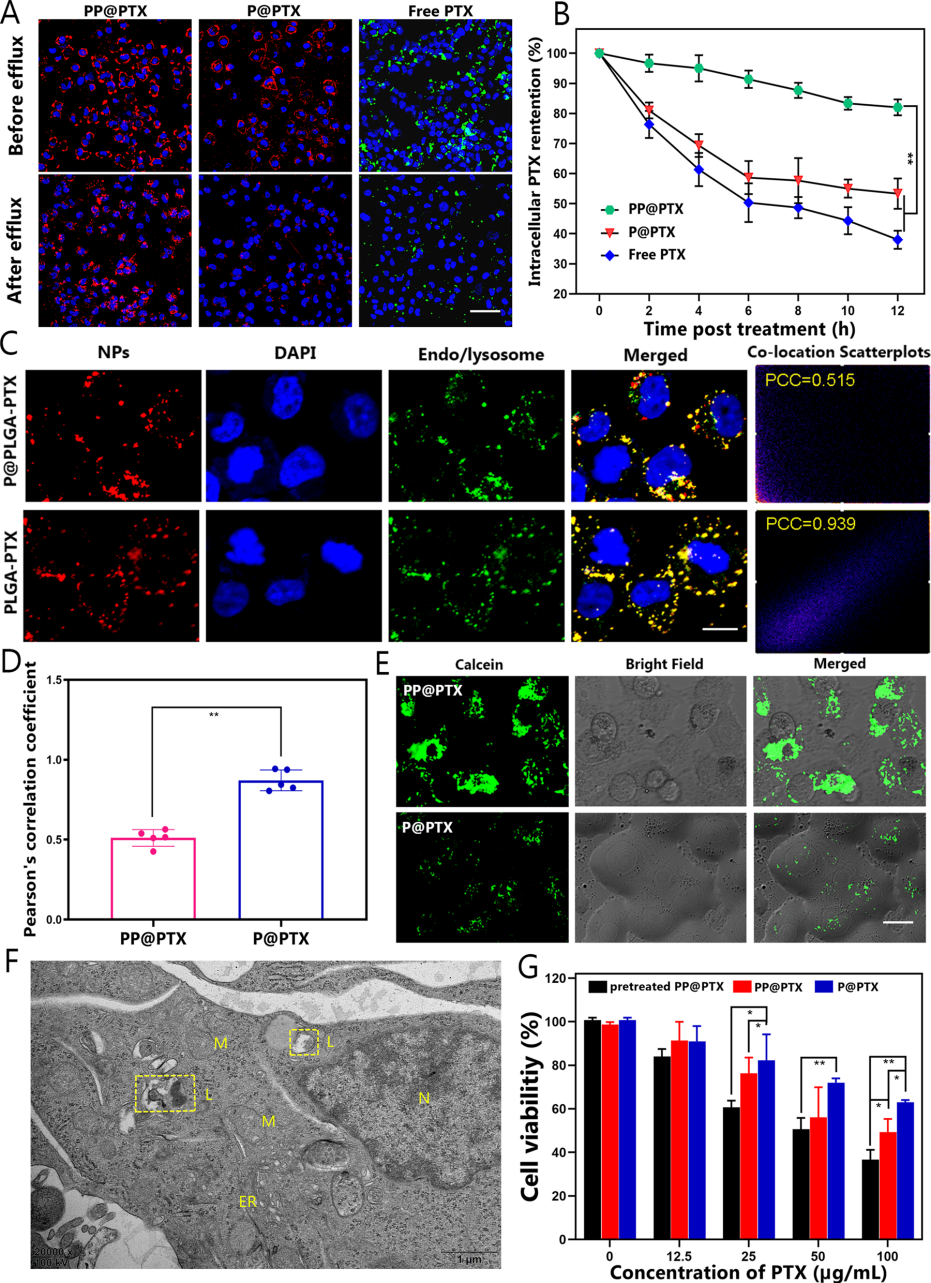
Intracellular accumulation and endo/lysosomal escape capability of the PEI-modified nanoparticle. **A** Intracellular PTX accumulation of MCF-7/Taxol cells coincubated with PP@PTX, P@PTX and free PTX before and after drug efflux. Blue fluorescence shows nuclei from DAPI; red fluorescence shows DiI-labeled PP@PTX NPs and P@PTX NPs; green fluorescence shows FTIC-labeled free PTX. The scale bar is 100 μm. **B** Intracellular PTX retention in MCF-7/Taxol cells at various times after DiI-labeled PP@PTX, P@PTX and free FITC-PTX treatment. **C** The colocalization of NPs and endo/lysosomes in MCF-7/Taxol cells after coincubation with DiI-labeld PP@PTX and P@PTX. Lysotracker stains the endo/lysosomes (green), and the NPs trapped in endo/lysosomes are labeled as yellow dots. Colocation scatterplots of PP@PTX and P@PTX vs endo/lysosomes are analyzed by Image J. The scale bar is 25 μm. **D** Corresponding Pearson’s correlation coefficient (PCC) values of PP@PTX and P@PTX vs endo/lysosomes after 6 h coincubation, n = 5 per group. **E** Integrity of endo/lysosomal membrane with calcein after conincubation with PP@PTX and P@PTX. The scar bar is 25 μm. **F** Bio-TEM images of MCF-7/Taxol cells incubated with PP@PTX for 6 h. The ROI (yellow square in the image) reveals the membrane rupture and burst of lysosomes. *L* lysosome, *N* nucleu, *M* mitochondria, *ER* endoplasmic reticulum. **G** Relative cell viability of MCF-7/Taxol cells after incubation with P@PTX, PP@PTX, and pretreated PP@PTX (pre-immersed in PBS at pH 6.3 for 2 h) at different concentrations for 24 h. All data are presented as mean ± SD, *p < 0.05, **p < 0.01

Considering that MDR is mostly understood as primarily involving the exportation of drugs by efflux pumps, we hypothesized that high-efficiency intracellular uptake and preservation of NPs were central to improving the clinical outcomes of chemotherapy. Thus, the properties of PEI-modified NPs to assist chemotherapeutic agents in overcoming MDR through escape from endo/lysosomal sequestration and hydrolytic degradation were further studied. To observe intracellular drug retention, MCF-7/Taxol cells were first treated with NPs with PEI modification, NPs without PEI modification or free FITC-PTX, which was then replaced with an NP-free solution for follow-up observations. As shown in CLSM images (Fig. 2A), extensive NPs emitting red fluorescence were visible in the cytoplasm in MCF-7/Taxol cells in the PP@PTX group, compared with the P@PTX and free FTIC-PTX groups, where the PTX level decreased rapidly in the MCF-7/Taxol cells. This outcome suggested that there was a significantly slower drug efflux rate when the cells were coincubated with PP@PTX. The fluorescence intensities further revealed that the concentrations of intracellular PTX in MCF-7/Taxol cells treated with free FITC-PTX and P@PTX were only 38.0% and 53.3%, respectively, suggesting apparent drug efflux via P-gp. In contrast, the retention ratio of PTX increased significantly when the cells were treated with PP@PTX, reaching 82.0% PTX retention in MCF-7/Taxol cells. The foregoing results collectively indicated that PTX efficiently accumulated and retained in drug-resistant cells with the assistance of PEI-modified nanoagents (Fig. 2B and Additional file 1: Figure S10). To investigate the mechanism of drug retention enhancement that was probably mediated by endo/lysosomal escape, CLSM was subsequently employed to observe the colocalization of NPs and endo/lysosomes in MCF-7/Taxol cells after coincubation with PP@PTX and P@PTX. As depicted in Fig. 2C, most P@PTX NPs were trapped inside the endosome or lysosome which showed yellow fluorescence (due to the overlap of green and red dots). In contrast, distinct red and green signals were observed when analyzing the PP@PTX group, which indicated the successful endo/lysosomal escape of the NPs. The corresponding Pearson’s correlation coefficient (PCC) was determined to be markedly lower in the PP@PTX group than that in the P@PTX group, again suggesting a low degree colocalization of PP@PTX with endo/lysosomes (Fig. 2D) [53, 54]. A membrane-impermeable fluorophore, calcein, which displays punctuated fluorescence patterns when it is entrapped in endo/lysosomes, was used to assess the integrity of endo/lysosomal membrane. However, a spread and bright fluorescence were detected after coincubation with PP@PTX, implying endosomal/lysosomal membrane was disrupted and calcein escaped. Since phospholipids with negative charges are commonly believed to form outer layers of endosomal membranes, cations from PEI modified NPs trapped by endosomes may interact with anionic phospholipids to trigger a ‘‘flip-flop” mechanism, where charge-neutralized ion pairs will lead to nonlamellar phase changes and endo/lysosomal membrane destruction. Furthermore, the biological TEM images of the cells before and after incubation with PP@PTX (preincubation and 3, and 6 h after incubation) were used to visualize the process of endo/lysosomal destruction. Oval-shaped endosomes or lysosomes were charged with electron-dense material before treatment with the NPs. Some of these organelles displayed morphological changes after coincubation with PP@PTX, becoming swollen with partial membrane rupture as depicted in Fig. 2E and Additional file 1: Figure S11 [55]. These results proved the endo/lysosomal destruction capability owing to the proton sponge effect of the PEI-modified NPs, thereby enhancing the release of the cargoes. In light of the excellent intracellular accumulation and endosomal escape capability of the PEI-modified NPs, we hypothesized that PP@PTX allowed more anticancer drugs to be retained in the MDR cells to increase the cytosolic concentration of PTX, and thus mitigating drug resistance caused by efflux pumps [56]. The cytotoxicities of PP@PTX and P@PTX with different PTX concentrations in MCF-7/Taxol cells was then studied. PP@PTX was first immersed in PBS at pH 6.3 for 2 h to simulate the acidity of the tumor microenvironment (TME). After coincubation with different formulations (pretreated PP@PTX, P@PTX, and PP@PTX) for 24 h, the viability of the MCF-7/Taxol cells was assessed by using the CCK-8 assay. Notably, the decrease in cell viability depended on the increased drug concentration, which decreased more sharply in the pH-treated PP@PTX group compared with the other groups. This result was speculated to arise because fact that PEI-modified NPs more readily trigger destabilization of the endosomal membrane by absorbing numerous protons in the lower pH environment, leading to better intracellular retention and cytotoxic effects to reverse MDR in vitro (Fig. 2F) [57].

### In vitro and vivo of targeting and penetrating abilities

**Fig. 3:**
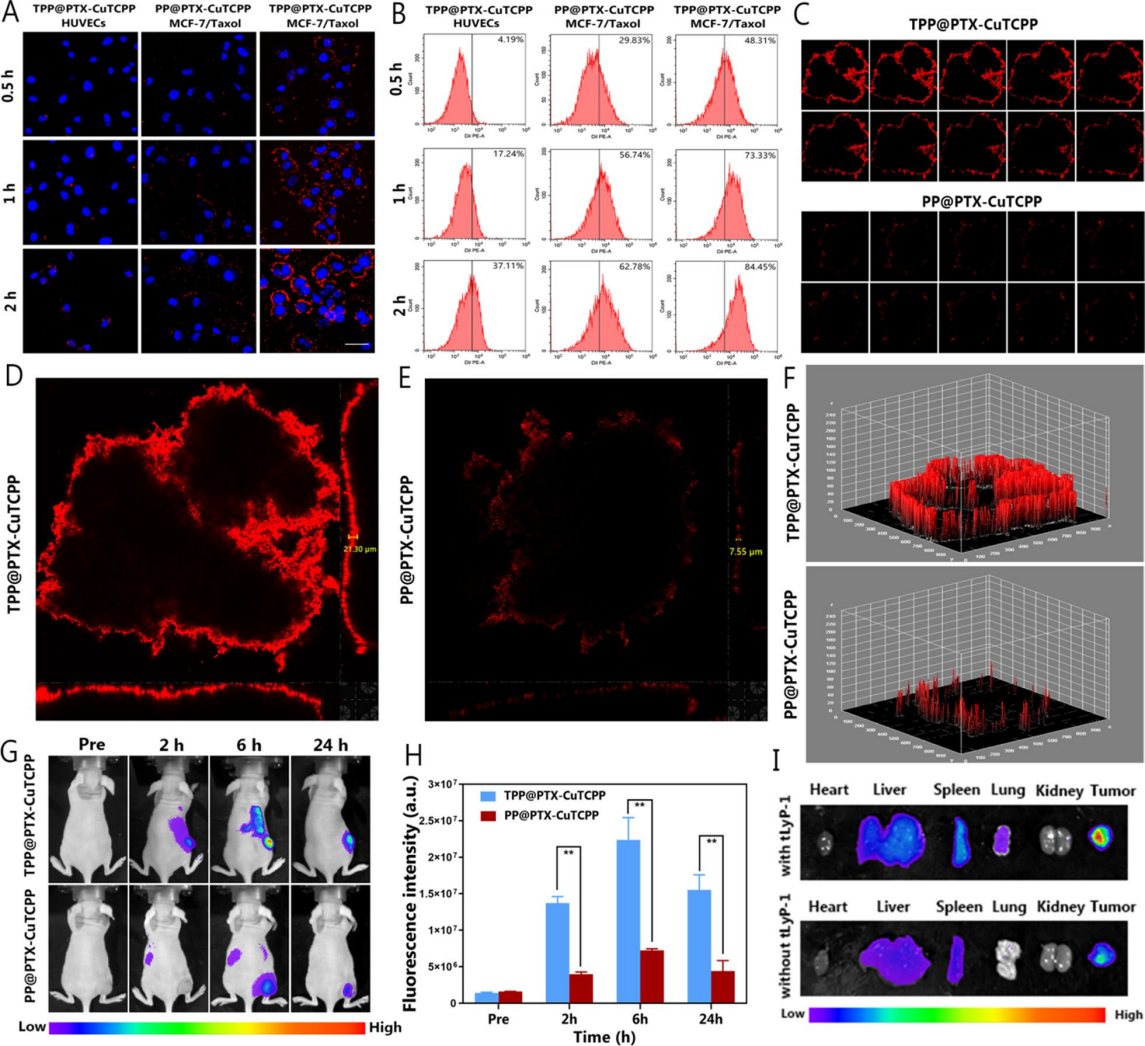
In vitro and in vivo targeting and penetrating behavior endowed by tLyP-1 peptide. **A** CLSM images of the MCF-7/Taxol and HUVECs cells coincubated with TPP@PTX-CuTCPP and PP@PTX-CuTCPP for 0.5 h, 1 h, and 2 h, respectively. Red fluorescence shows DiI-labeled PP@PTX and P@PTX NPs; blue fluorescence represents nuclei attributing to DAPI. The scale bar is 50 μm. **B** Flow cytometry analysis of MCF-7/Taxol and HUVECs cells treated with DiI-labeled TPP@PTX-CuTCPP and PP@PTX-CuTCPP for 0.5 h, 1 h, and 2 h, respectively. **C** Multiple level scan started at the bottom of the spheroid at 2 μm intervals for the penetration of DiI-labeled TPP@PTX-CuTCPP NPs and PP@PTX-CuTCPP NPs. **D**, **E** Quantitative analysis of the penetration depth of TPP@PTX-CuTCPP (left) and PP@PTX-CuTCPP (right) in three-dimensional tumor spheroid models. **F** Surface plots based on three-dimensional reconstruction of the MCF-7/Taxol spheroid models incubated with TPP@PTX-CuTCPP NPs (upper) or PP@PTX-CuTCPP NPs (lower) for 6 h. **G** In vivo NIR fluorescence images of tumors in MCF-7/Taxol tumor-bearing mice after intravenous injection of TPP@PTX-CuTCPP and PP@PTX-CuTCPP at different time points. **H** Corresponding fluorescence intensity (tumor:background) of MCF-7/Taxol tumor-bearing mice administered two types of NPs. Data are presented as mean ± SD, n = 3 per group. **I** Ex vivo fluorescence images of major organs and tumors dissected from mice 24 h postinjection of NPs with and without tLyP-1

To demonstrate that tLyP-1 could enhance the targeting ability of the nanoplatform, the intracellular behavior of TPP@PTX-CuTCPP was visualized by CLSM. As shown in Fig. 3A, increased DiI-labeled TPP@PTX-CuTCPP emitting red fluorescence aggregated around the cytomembrane of the MCF-7/Taxol cells that overexpressed NRP-1 after coincubation with monolayer cells for 0.5, 1, and 2 h. However, fewer NPs gathered around the cells in the DiI-labeled PP@PTX-CuTCPP group. The role of tLyP-1 in guiding TPP@PTX-CuTCPP to NRP-1 receptor-rich tumor cells was verified by incubating with NRP-1 receptor-negative HUVECs as a control. The results demonstrated that the number of aggregated TPP@PTX-CuTCPP NPs decreased markedly in HUVECs compared with those in MCF-7/Taxol cells. The flow cytometry results, which revealed stronger fluorescence in the MCF-7/Taxol cells after coincubation with TPP@PTX-CuTCPP were, in accordance with the CLSM images (Fig. 3B). To study the penetration mechanism of NPs functionalized by THPP, a three-dimensional tumor spheroid model was successfully established in this study which can better simulate the TME of solid tumors due to the high cell density, increased interstitial pressure, and heterogeneous tumor perfusion in the spheroid model. After coincubation with DiI-labeled TPP@PTX-CuTCPP or PP@PTX-CuTCPP for 6 h, we found that the red fluorescence from TPP@PTX-CuTCPP was more evidently distributed in the tumor spheroids than that from PP@PTX-CuTCPP (Fig. 3C, Additional file 1: Figures S12, S13). Furthermore, tLyP-1 enabled these versatile nanosystems to distribute throughout the whole spheroid; however, PP@PTX-CuTCPP adhered to only the margin of the tumor spheroid via three-dimensional reconstruction. Quantitative analysis revealed that the penetration depth of TPP@PTX-CuTCPP (21.30 μm) reached to the core of the tumor spheroid, which was 2.82-fold greater than that of PP@PTX-CuTCPP with a penetration depth of only 7.55 μm (Fig. 3D–F). The remarkable variation in metabolism between normal tissue and tumor tissue leads to the different physical and chemical properties from the internal environment of solid tumors, such as high pressure, low pH, and inhomogeneous microvessels. Owing to the satisfactory targeting and penetrating performance, we speculated that the tLyP-1 peptide could facilitate the transportation of the NPs into the core of tumor spheroids possibly favoring the MDR reversal. Additionally, we used MCF-7/Taxol tumor-bearing mice to validate the in vivo targeting ability via FL imaging and quantitative analysis. The fluorescence intensity at the tumor location in the TPP@PTX-CuTCPP group was significantly stronger than that in the PP@PTX-CuTCPP group at the predetermined time intervals. The FL signal peaked at 6 h after the administration of DiR-labeled TPP@PTX-CuTCPP, which also exhibited relatively longer retention in the tumor region than was found in the nontargeted group (Fig. 3G, H). Then, the tumors and major organs (heart, liver, spleen, lung, and kidney) were next harvested to determine the ex vivo biodistribution of the NPs 24 h postinjection. The results revealed that both two types of NPs were accumulated in the liver and spleen due to phagocytosis by the reticuloendothelial system (RES). However, greater accumulation of the NPs in tumor tissue was found in TPP@PTX-CuTCPP group as shown in Fig. 3I and Additional file 1: Figure S14. Additionally, the ultrathin frozen tumor sections were prepared for CLSM analyses. The TPP@PTX-CuTCPP group contained more red dots in tumor tissue than did the PP@PTX-CuTCPP (Additional file 1: Figure S15). These results demonstrated again the desirable targeting feature of tLyP-1. In particular, we found that PP@PTX-CuTCPP NPs mainly gathered in microvessels, which were stained by CD31, with little extravasation. However, TPP@PTX-CuTCPP NPs spread into the extravascular tumor tissue and reached into a deeper area (Additional file 1: Figures S16, S17). This evidence firmly revealed that tLyP-1 functionalized NPs with active tumor-penetrating properties are promising for anticancer drug carriage.

### Scavenging effect of GSH and PAI in vitro and in vivo

**Fig. 4:**
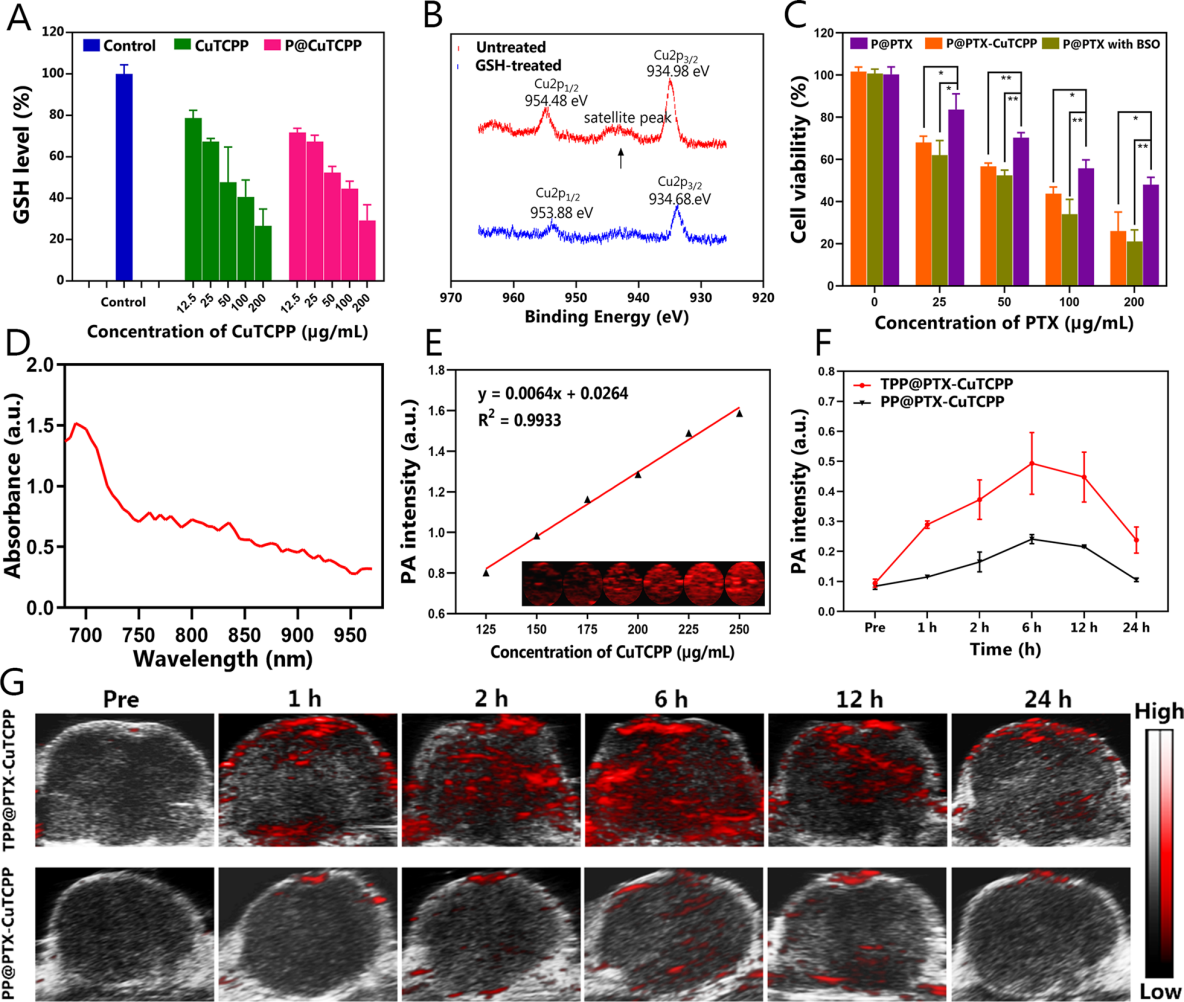
GSH depletion efficiency and photoacoustic contrast-enhanced imaging. **A** Residual GSH content in aliqutos determined by GSH assay kit after different corresponding concentration in P@CuTCPP and CuTCPP. **B** Detailed XPS spectrum of CuTCPP in the scanning regions of Cu before and after treating with GSH solution. The black arrow shows the position of the satellite peak. **C** Relative cell viability of MCF-7/Taxol cells after incubation with P@PTX, P@PTX-CuTCPP and P@PTX with BSO for 24 h at different concentrations. **D** The detection of optimal excitation wavelength by full spectrum scanning from 680 to 970 nm in PAI system. **E** In vitro PAI images and PAI values of TPP@PTX-CuTCPP at different CuTCPP concentrations. **F** Average photoacoustic intensity at tumor region after intravenous injection of NPs at different time intervals, n = 3 per group. **G** In vivo PAI images of tumors in MCF-7/Taxol tumor-bearing mice after intravenous injection TPP@PTX-CuTCPP and PP@PTX-CuTCPP at various time intervals. All data are presented as mean ± SD, *p < 0.05, **p < 0.01

It is anticipated that Cu^2+^-based nanoagents can cause an imbalance in the the GSH-involved redox metabolism equilibrium, further relieveing MDR by reshaping the TME. The proposed mechanism was first studied in GSH solution and tested using 5,5′-dithiobis-2-nitrobenzoic acid (DTNB), a tracker for relative intracellular GSH levels by monitoring the variation in UV–vis absorbance intensity of the intermediate product at 405 nm, after mixing with NPs delivering CuTCPP, free CuTCPP, and deionized water at varying concentrations for 24 h. As depicted in Fig. 4A, there was comparative GSH-depletion efficiency from the free CuTCPP and P@CuTCPP groups containing equivalent doses of CuTCPP, which suggested that the nanosystem comprising CuTCPP inherited the potent GSH scavenging capability of CuTCPP. In addition, the valence state of Cu was determined by XPS, which enables surface analysis with a quantitative accuracy of 90–95% [58, 59]. The binding energies of Cu2p_1/2_ (954.48 eV) and Cu2p_3/2_ (934.98 eV) in CuTCPP, which can be assigned to Cu^2+^ peaks, were observed before treatment with GSH. The satellite peak at 942.98 eV between Cu2p_3/2_ and Cu2p_1/2_ in the paramagnetic chemical state also provided credible evidence for the presence of the Cu^2+^ valence state. Interestingly, the binding energies of Cu2p_3/2_ and Cu2p_1/2_ shifted slightly to 953.88 eV and 934.68 eV, respectively, and a decrease in the original satellite peak was found after treating GSH solution. Additionally, we further analyzed the sample by comparing the integrated peak areas. The atomic percentage of Cu^2+^ decreased from 96.40 to 30.66% after treatment with GSH. These results firmly suggested the partial reduction in the copper valence state from + 2 to + 1 during the reaction (Fig. 4B and Additional file 1: Figure S18) [60, 61]. Subsequently, MCF-7/Taxol cells were used to detect intracellular GSH levels after coincubation with P@CuTCPP, and PLGA in combination with l-buthionine sulfoximine (l-BSO), an inhibitor of GSH synthesis. The results revealed that while l-BSO inhibited the synthesis of intracellular GSH most remarkably, P@CuTCPP also exhibited moderate GSH-depletion performance among the three groups (Additional file 1: Table S1). Inspired by the considerable GSH depletion by CuTCPP, we assessed the cytotoxicity of PTX in the presence and absence of CuTCPP to verify whether the GSH scavenging effect sensitized the chemotherapy against MDR cells. Notably, the viability of MCF7/Taxol cells treated with P@PTX-CuTCPP or P@PTX combined with a nontoxic dose of l-BSO was significantly lower than that of cells treated with P@PTX only, as shown in Fig. 4C. Recent studies have demonstrated that Cu-involved nanomaterials have promising prospect in tumor theranostics [60], the antibacterial use [62] and bioimaging due to the generation of reactive oxygen species from Cu^2+^. In present study, although the low-dose use of Cu^2+^ (up to 0.1 mM) alone failed to conquer tumor cells, we found that CuTCPP can be a synergist to enhance chemotherapy efficacy after encapsulaton into nanosystems. Light absorption in the NIR region is a prerequisite for NIR-induced imaging. To investigate the potential of TPP@PTX-CuTCPP as a PAI contrast agent for tumor monitoring, an in vitro gel experiment was initially performed. An excitation wavelength of 690 nm was selected as the optimal wavelength for enhanced PAI in the following experiments after full spectrum scanning in photoacoustic system (Fig. 4D). The results showed that the photoacoustic intensities strengthened after increasing the CuTCPP concentration from 125 to 250 µg mL^−1^. Quantitative analysis revealed that the photoacoustic intensity of the NPs was concentration-dependent with a good linear relationship (R^2^ = 0.99) corresponding to contrast-enhanced imaging, which indicated that the imaging features of TPP@PTX-CuTCPP were triggered by laser irradiation (Fig. 4E). Subsequently, MCF-7/Taxol tumor-bearing mice were used to confirm PAI capability after injection of TPP@PTX-CuTCPP or PP@PTX-CuTCPP solution. The photoacoustic images were then obtained at predetermined time points (preinjection, and 1, 2, 6, 12, and 24 h postinjection), and corresponding quantitative photoacoustic intensity analysis was performed. The results showed that there were negligible photoacoustic signals at the tumor region in both group before NP injection. After injection with TPP@PTX-CuTCPP, the photoacoustic signal at the tumor region gradually increased and reached a peak value approximately 6 h post injection which was dramatically stronger than that observed in the PP@PTX-CuTCPP group. With increasing time after post injection, the tumor photoacoustic value started to decrease due to the clearance of NPs; nevertheless, the signal in the TPP@PTX-CuTCPP group was still significantly stronger than that observed in the PP@PTX-CuTCPP group owing to the targeting behavior of tLyP-1 (Fig. 4F, G). These results confirmed that the TPP@PTX-CuTCPP could effectively accumulate in tumor tissue and serve as a contrast-enhanced agent for PAI.

### Cytotoxicity and anticancer effects in vitro

**Fig. 5:**
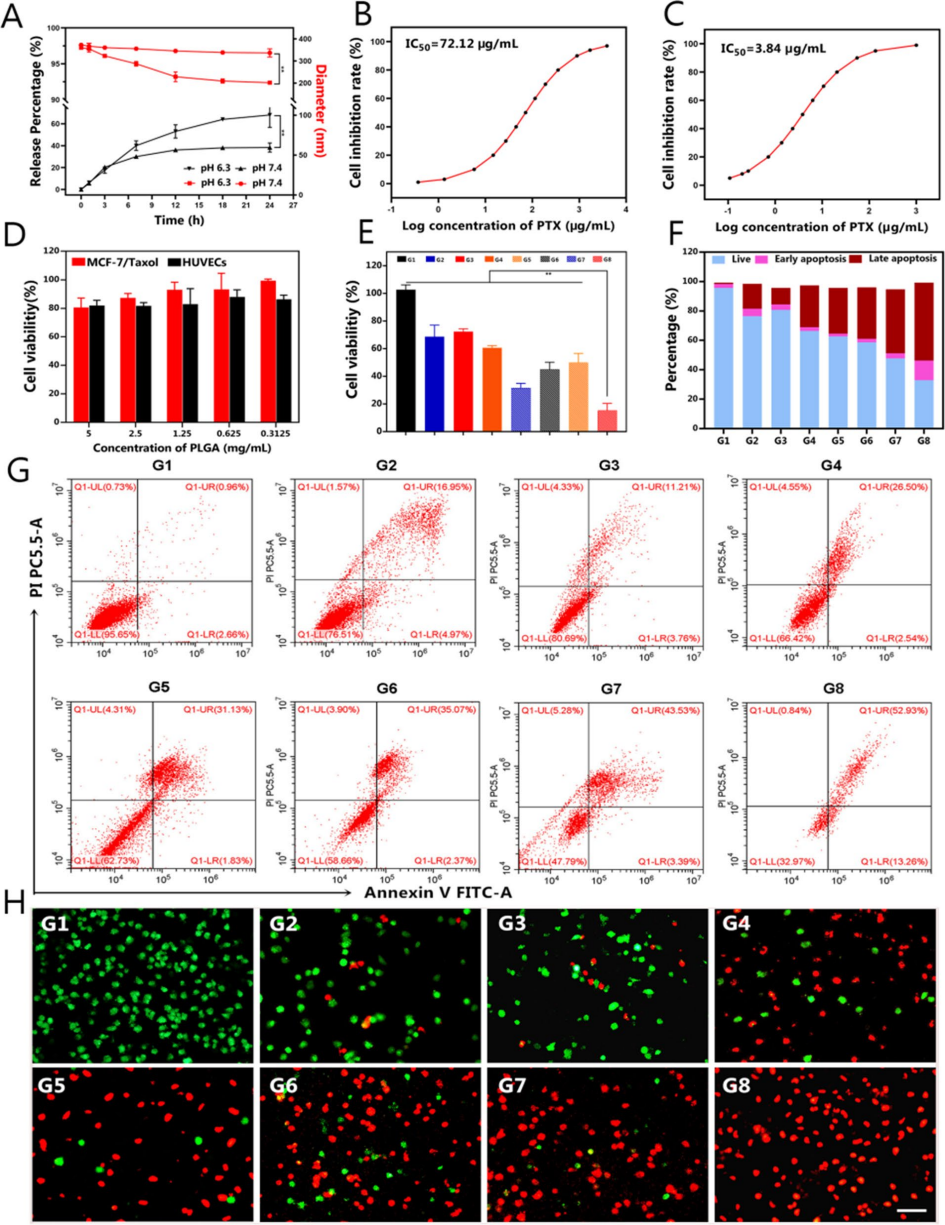
Synergistic chemotherapy efficacy of the various nanoformulations in vitro. **A** pH-responsive drug release changes and time-dependent changes of the size of TPP@PTX-CuTCPP under different pH values (6.3 and 7.4) at various time intervals. **B**, **C** IC_50_ curves of PTX against MCF-7/Taxol drug resistant cell line and MCF-7 parental cell line. **D** Biocompatibility of TPP@CuTCPP (PTX-free) against MCF-7/Taxol and HUEVCs cells at varied concentrations for 24 h treatment. **E** Relative cell viability of MCF-7/Taxol cells after coincubation with corresponding formulations for 24 h. The concentration of PTX is 50 μg/mL, n = 3 per group. **F**, **G** Flow cytometric analysis on the apoptosis levels of MCF-7/Taxol cells of after corresponding treatments. **H** CLSM images of MCF-7/Taxol cells stained by calcein AM and PI after different corresponding treatments. Green fluorescence represents live cell, red fluorescence represents dead cell. The scale bar is 100 μm. **G1**–**G8** represent control, free PTX, P@PTX, P@PTX-CuTCPP, PP@PTX, TPP@PTX, PP@PTX-CuTCPP, and TPP@PTX-CuTCPP, respectively. All data are presented as mean ± SD, **p < 0.01

The release process of PTX from the NPs was quantitatively investigated by soaking the samples in buffer solution at different pH values (pH = 6.3 and 7.4) to simulate the intracellular environments. Approximately 68.1% of the loaded PTX was released from TPP@PTX-CuTCPP immersed at pH 6.3 after 24 h, higher than the 38.3% released from these NPs in solution at physiologic pH (7.4) (Fig. 5A). This difference may be explained by the responsiveness of the NPs to a weakly acidic environment in which the PLGA shell layer gradually disassembles, triggering the high-efficiency release of PTX from the delivery system [63]. Interestingly, time-dependent changes in NP size at varying time intervals were observed by DLS and TEM. An increasingly smaller size of NPs was detected over time, as presented in Fig. 5A and Additional file 1: Figure S19, which corresponded to the PTX release ratio. Due to the impressive drug release behavior of the NPs, the in vitro cytotoxic and anticancer effects were next investigated. The IC50 values of PTX against the parental cells (MCF-7) and drug-resistant cells (MCF-7/Taxol) were initially determined using the CCK-8 assay. An 18.78-fold higher IC_50_ value was observed in MCF-7/Taxol cells than that in MCF-7 cells, suggesting the successful establishment of a PTX-resistant cell line (Fig. 5B, C). Furthermore, the cytotoxicity of TPP@CuTCPP (PTX-free) was evaluated in MCF-7/Taxol cells and HUVECs to assess the biosafety of the drug-delivery vehicle and verify that the anticancer effects originate from PTX. TPP@CuTCPP exerted only a negligible effect on the survival of MCF-7/Taxol cells and HUVECs even at PLGA concentrations as high as 5 mg mL^−1^, as shown in Fig. 5D, displaying favorable low toxicity and biocompatibility as a drug carrier for further application. To study the anticancer effects of the various NPs after carrying PTX, the viabilities of MCF-7/Taxol cells treated with the following eight compounds for 24 h were evaluated: G1, control; G2, free PTX; G3, P@PTX; G4, P@PTX-CuTCPP; G5, PP@PTX; G6, TPP@PTX; G7, PP@PTX-CuTCPP; and G8, TPP@PTX-CuTCPP. As shown in Fig. 5E, limited cytotoxicity was detected in the group of free PTX and P@PTX groups, which could be ascribed to the drug resistance speciality from MCF-7/Taxol cells. When PEI, CuTCPP, or tLyP-1 was added to the nanosystem, the cell viability markedly decreased to different degrees. Of note, TPP@PTX-CuTCPP exhibited significantly greater cytotoxicity than the other treatments, demonstrating the best anticancer efficacy. The coincubation of MCF-7/Taxol cells with various concentrations of TPP@PTX-CuTCPP was then investigated. Cell viability decreased with increasing TPP@PTX-CuTCPP concentrations, showing that the therapeutic effect was concentration-dependent (Additional file 1: Figure S20). Flow cytometry was performed to quantitatively analyze cell apoptosis induced by the NPs. The determined percentage of apoptotic and live cells was similar to that found in the CCK-8 assay (Fig. 5F, G). Moreover, to visually evaluate the synergistic effects of the various formulations, we stained the cells with PI and calcein AM to determine the dead and live cells, which also confirmed that TPP@PTX-CuTCPP induced maximum cell death (Fig. 5H). We presumed that amplified antitumor efficacy was attributed to simultaneously improved drug internalization and retention by tLyP-1 and PEI. On the other hand, GSH scavenge synergized increased intracellular chemotherapeutic agents, further enhancing cytotoxicity of PTX. To prove that the combined reversal strategies involved synergistic instead of an additive effects, the combination indexes (CIs) of different nanoformulations were calculated by the Chou–Talalay method [64]. As shown in Additional file 1: Table S2, nanoformulations involving one or two strategies showed moderate synergism with PTX; notably, the CI of the nanoformulation with triple strategies (TPP@PTX-CuTCPP) was 0.201, which indicated the strong synergism. These results jointly indicated that the multifunctional NPs possessing tumor homing-penetrating, endo/lysosomal escape and GSH depletion capabilities can synergistically enhance the anticancer efficacy of PTX for the reversal of MDR.

### Anticancer therapy in vivo

**Fig. 6:**
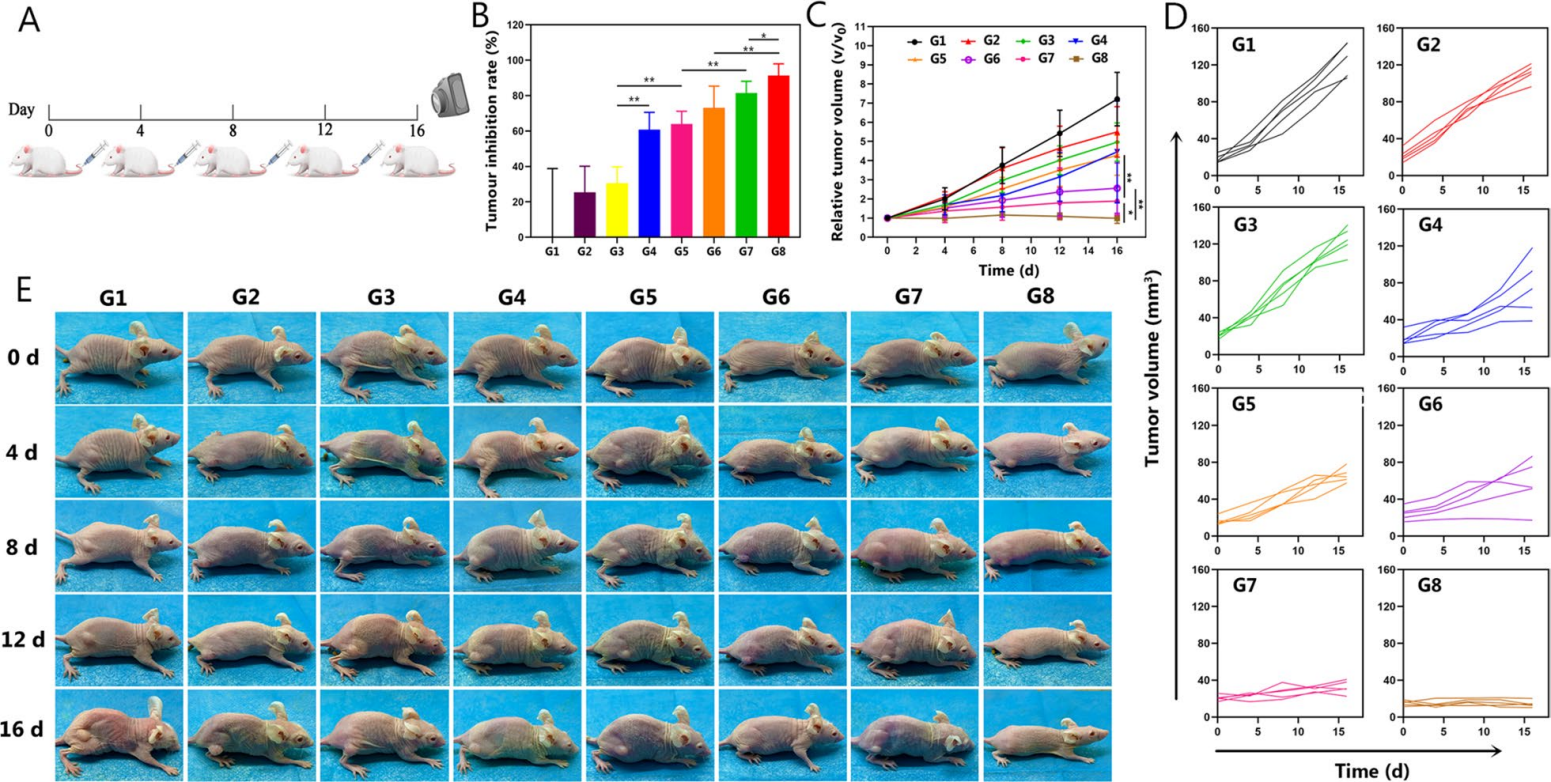
Evaluations on the combined effects for enhancing chemotherapy-based anti-tumor efficacy in vivo. **A** Schematic illustration of the treatment regimen. All groups were intravenously administered with different NPs on 0 day, 4 days, 8 days, 12 days. **B** Tumor inhibition rates of eight groups after various treatments, n = 5 per group. **C** Relative tumor growth curves of different groups of MCF-7/Taxol tumor-bearing mice, n = 5 per group. **D** Tumor growth curves of each mouse in G1–G8. **E** Representative digital pictures of MCF-7/Taxol tumor-bearing mice of eight groups during 16 days period after intravenous administration of different formulations. G1–G8 represent control, free PTX, P@PTX, P@PTX-CuTCPP, PP@PTX, TPP@PTX, PP@PTX-CuTCPP, and TPP@PTX-CuTCPP, respectively. All data are presented as mean ± SD, *p < 0.05, **p < 0.01

**Fig. 7:**
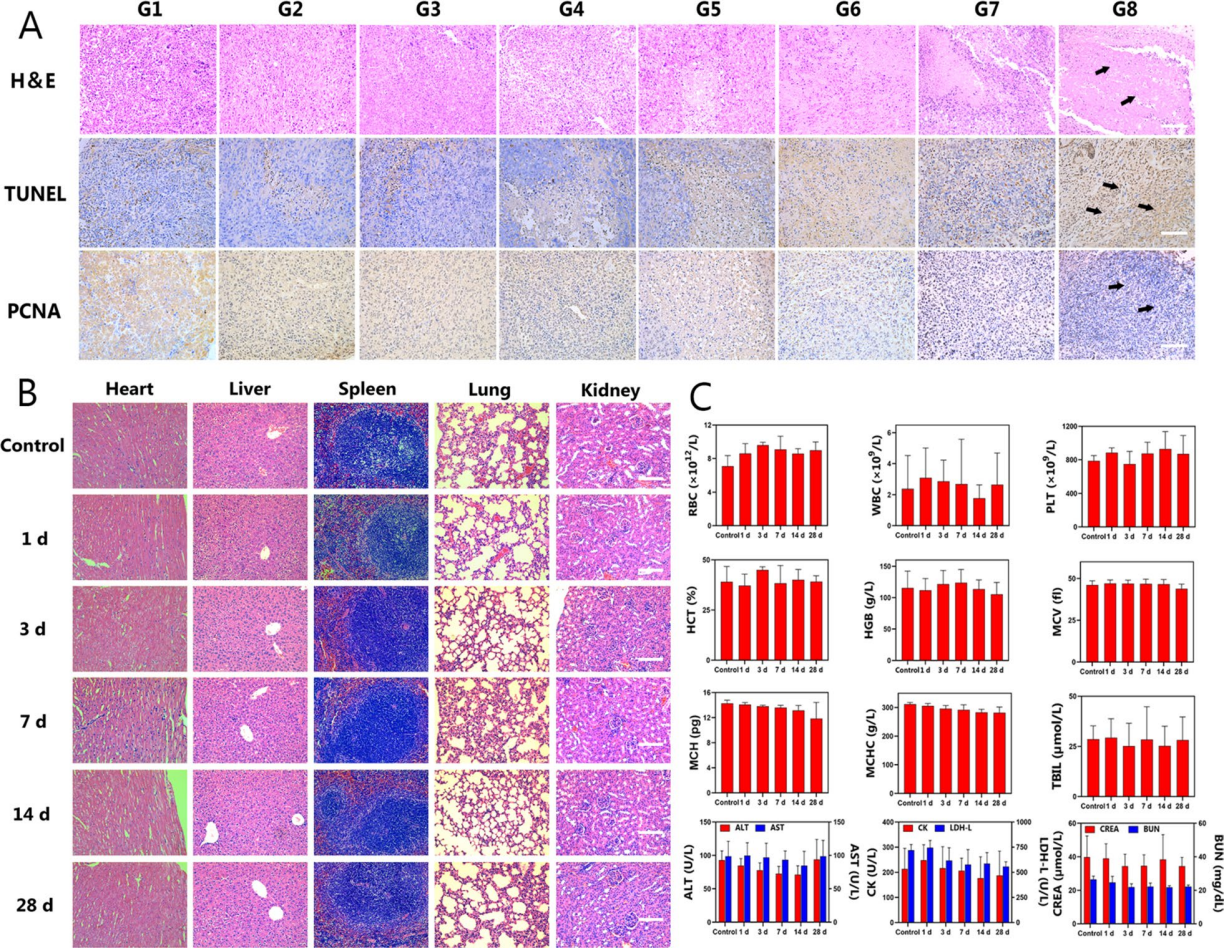
Pathological and blood biochemistry results of tumors and major organs in vivo. **A** H&E, TUNEL, and PCNA staining on tumor sections from MCF-7/Taxol tumor-bearing mice after various treatments. The scale bar is 50 μm. **B** H&E staining of the heart, liver, spleen, lungs, and kidneys in mice sacrificed at different time intervals (Control, 1 days, 3 days, 7 days, 14 days, 28 days) after intravenous injection of TPP@PTX-CuTCPP NPs. The scale bar is 100 μm. **C** Blood biochemistry and complete blood analysis of Kunming mice sacrificed at different time intervals (Control, 1 days, 3 days, 7 days, 14 days, 28 days) after intravenous injection of TPP@PTX-CuTCPP NPs. Data are shown as mean ± SD, n = 4 per group. G1–G8 represent control, free PTX, P@PTX, P@PTX-CuTCPP, PP@PTX, TPP@PTX, PP@PTX-CuTCPP, and TPP@PTX-CuTCPP, respectively. Black arrows represent cell necrosis, apoptotic cells and proliferative cells in H&E, TUNEL and PCNA sections, respectively. All data are presented as mean ± SD

The chemotherapeutic efficacy in vivo was evaluated by monitoring tumor growth and measuring the changes in tumor volume for 16 days in MCF7/Taxol tumor-bearing mice (Fig. 6A). The tumor-bearing mice were randomly divided into eight groups for different treatments (n = 5): G1, control; G2, free PTX; G3, P@PTX; G4, P@PTX-CuTCPP; G5, PP@PTX; G6, TPP@PTXP; G7, PP@PTX-CuTCPP; and G8, TPP@PTX-CuTCPP. As shown in Fig. 6C, D, the mice treated with various compounds exhibited slowed tumor growth to different degrees during 16 days of observation and had the highest antitumor effects after treatment with G8. In addition, it was noteworthy that G8 was the only group that not only suppressed the rate of the tumor growth, but also shrank the tumor volume at the end of treatment (Fig. 6E). Furthermore, the tumor inhibition rate dramatically increased by 91.4% in the G8 compared with that in the control group at 16 d post injection, suggesting an almost tumor eradication (Fig. 6B). In contrast, limited tumor inhibition was observed in the control group and the G2, which was similar to the results found in the in vitro test. Thus, we speculated that the “combo” nanoagent acting on different mechanisms of drug resistance through multiple synergistic strategies would provide the best efficacy for the reversal of MDR. PCNA, TUNEL, and H&E staining of the tumor sections were further conducted to confirm the chemotherapeutic efficacy of the multifunctional NPs. H&E staining showed that severe apoptosis and necrosis were found in the G8, while only moderate damage appeared in the G7, G6, and G5. The PCNA and TUNEL assays had the similar trends. Representative apoptosis-positive cells are indicated by dark-brown nuclei in the TUNEL assay. Of note, the most positive index was observed in the group receiving TPP@PTX-CuTCPP, where the tumors were almost eliminated without relapse at the end of the treatment period. PCNA immunochemical staining of the tumors showing the in vivo proliferative activities was consistent with the results of the TUNEL assay where proliferative cells were stained into brown (Fig. 7A). The results of TUNEL and PCNA assays collectively demonstrated that TPP@PTX-CuTCPP has greater MDR reversal efficacy than a single strategy. Additionally, H&E staining of the major organs (including the heart, liver, spleen, lungs, and kidneys) showed no pathological damage or inflammatory lesions during the in vivo treatments in all groups (Additional file 1: Figure S21).

### Biosafety assay of TPP@PTX-CuTCPP in vivo

The in vivo biosafety of the TPP@PTX-CuTCPP in both the short and long terms (1 day, 3 days, 7 days, 14 days, and 28 days of post injection) was evaluated by histopathological and hematological analyses. H&E staining of the main organs indicated no histopathological changes or an abnormal inflammatory response in the all groups compared to the control group (Fig. 7B). Meanwhile, blood biochemical indexes showed no evident increase in the levels of indexes compared with the control group, indicating that the chemotherapy based on TPP@PTX-CuTCPP has good biocompatibility, making it fesible for future clinical translation (Fig. 7C). The body weight of the mice was also monitored during the period of therapy as shown in Additional file 1: Figure S22. There were negligible differences found in all groups, suggesting that the anticancer effects carried by the nanosized delivery system induced undetectable side effects to mice at the tested dose.

### Combined immunochemotherapy and immune responses in vivo

**Fig. 8:**
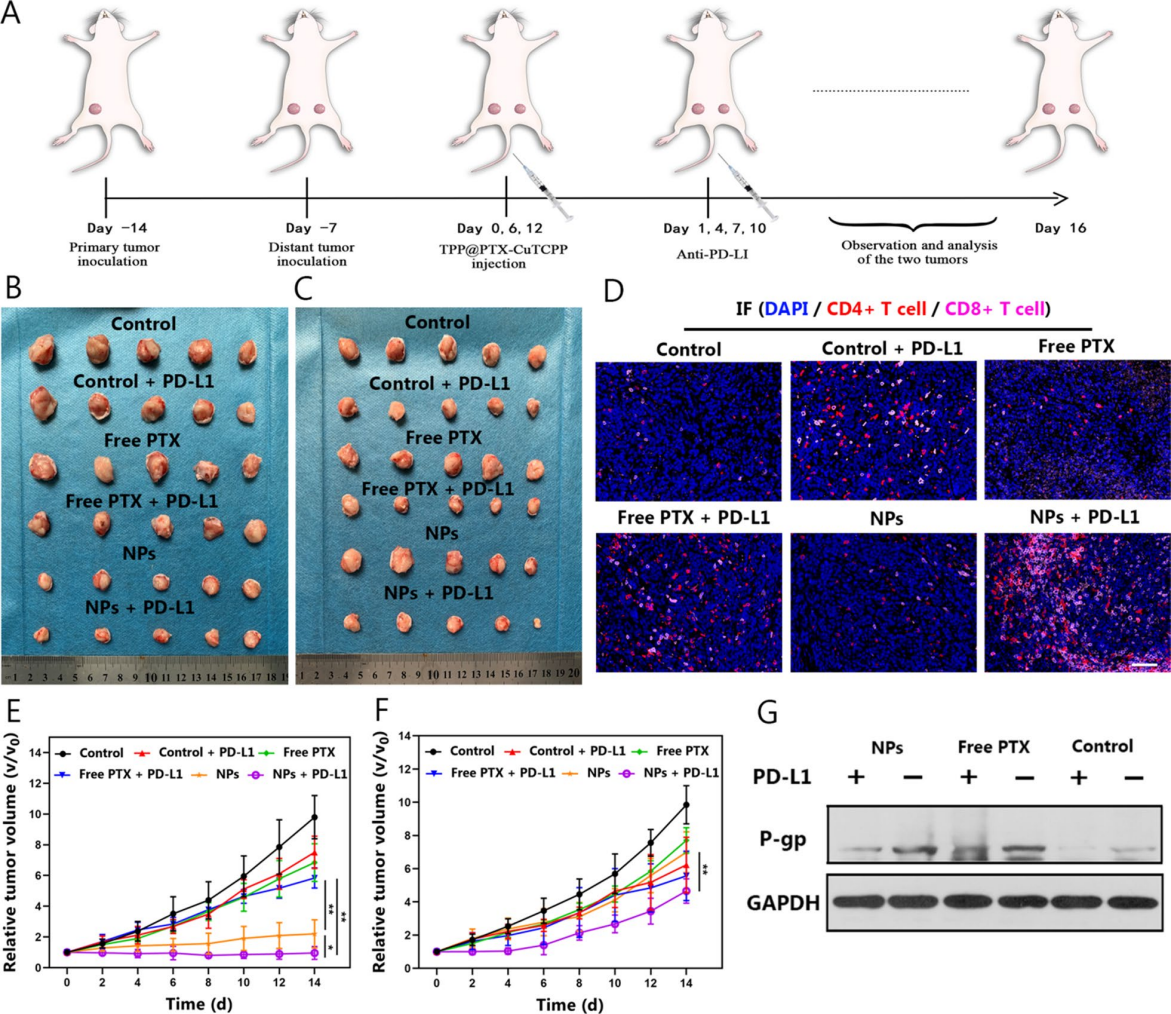
Anticancer efficacy and systemic immune responses initiated by immunochemotherapy in vivo. **A** Schematic illustration of the in vivo treatment design. **B**, **C** Representative digital pictures of 4T1 tumors of different groups at the end of 16 days post-treatment. **D** Immunofluorescence images of CD4^+^ (red) and CD8^+^ (pink) T cells in the distant tumors. **E**, **F** Relative tumor growth curves of primary and distant tumors after various treatments, n = 5 per group. **G** Western blot for the detection of P-gp expression in groups with and without anti-PD-L1 treatment. The scale bar is 50 μm. All data are presented as mean ± SD, *p < 0.05, **p < 0.01

To assess the in vivo anticancer efficacy of the combination of multifunctional nanoagents with checkpoint blockade therapy and the immune reaction after treatment with anti-PD-L1, bilateral mimic tumor models (primary and distant) in BALB/c mice were established (Fig. 8A). In subsequent experiments, antitumor efficacy initiated by immunochemotherapy was firstly investigated. For the primary tumors, both TPP@PTX-CuTCPP and TPP@PTX-CuTCPP + anti-PD-L1 notably inhibited tumor growth within 16 days posttreatment notably, whereas, free PTX or free PTX plus anti-PD-L1 showed no appreciable effect on tumor growth (Fig. 8B, E). Moreover, TPP@PTX-CuTCPP plus anti-PD-L1 exhibited superior antitumor efficacy, which was probably derived from the synergistic effect of immunochemotherapy. For the distant tumors, due to the activation of antitumor immunity from anti-PD-L1 treatment, tumor growth in the control + PD-L1, free + PD-L1 and TPP@PTX-CuTCPP plus anti-PD-L1 groups was partly suppressed. Massive apoptosis and necrosis stained by H&E were found in the tumor area, which was consistent with the appearance of tumor growth as revealed in Fig. 8C, F and Additional file 1: Figure S23. However, there was no significant inhibitory effect on the distant tumors in the group without anti-PD-L1 treatment. Subsequently, we evaluated systemic immunity elicited by anti-PD-L1. Helper T cells (CD4^+^) and cytotoxic T lymphocytes (CD8^+^) play important roles in the regulation of adaptive immunity and destruction of targeted cancer cells, therefore, the level of tumor-infiltrating T cells in the mimic distant tumors were costained with CD3^+^, CD4^+^ and CD8^+^ T cells for further flow cytometry analysis. The results showed that the proportion of CD4^+^ T cells in the TPP@PTX-CuTCPP + anti-PD-L1 group was 25.45%, with a significant elevation compared with the control group (0.84%). On the other hand, the absolute percentage of CD8^+^ T cells in the combined immunotherapy group occupies 14.88%, with an improvement of nearly 5.3-fold in comparison with the control group (Additional file 1: Figure S24). Interestingly, a similar trend was found in tumor tissue slices, in which greatly enriched CD4^+^ and CD8^+^ T cells in the distant tumor were observed by the immunofluorescence assay (Fig. 8D). It was reported that helper T cells could produce pro-inflammatory, such as TNF-α, IL-6, and IFN-γ, all of which could enhance the activation of innate immune response. ELISA assay was next employed to evaluate the secretion levels of TNF-α, IFN-γ, IL-6 and IL-12 in the serum from the mice in all groups. Consistent with the activation of tumor- infiltrating T cells, the higher secretion levels mentioned above were observed in groups with anti-PD-L1 treatment, again implying a successful initiation of the systemic immune response (Additional file 1: Figure S25). To verify whether anti-PD-L1 could enhance cytotoxicity via regulating P-gp expression, western blot was performed to evaluate P-gp expression level in primary tumor. As depicted in Fig. 8G, the P-gp expression level obviously decreased after treating with anti-PD-L1 in comparison to that in the groups without anti-PD-L1 treatment, which may consequently lead to higher anticancer drug accumulation in tumor cells.

## Conclusion

In summary, we successfully developed a “combo” nanoscale medicine that exhibited a variety of synergistic strategies to enhance tumor chemotherapy aimed at overcoming MDR. tLyp-1-PEI functionalized NPs rapidly triggered tumor homing-penetration and endo/lysosomal escape effects after internalization by tumors. The PTX released from the cracked endo/lysosomes was expected to efficiently accumulate and be retained in MCF-7/Taxol cells to reverse MDR and inhibit the growth of tumors compared with the low efficacy of an equivalent dose of free PTX. On the other hand, by introducing CuTCPP into the nanosystem, allowed upregulated GSH in tumor cells to be scavenged, which might further render MDR cells more vulnerable to anticancer agents. Based on the NIR absorption from CuTCPP, TPP@PTX-CuTCPP with imaging potential is promising to serve as a PAI enhanced-contrast agent for theranostic guidance and visual monitoring in one step. Additionally, immune reaction and P-gp inhibition were observed by blocking the PD-1/PD-L1 interaction, indicating that checkpoint blockade therapy may contribute to combating chemotherapy resistance in breast cancer. Overall, the novel nanoagent in combination with immunotherapy may provide an attractive strategy for fighting against tumor drug resistance.

## Supplementary Information

**Additional file 1:** **Scheme S1.** The synthetic route of tLyP-1-PEI-PLGA. **Figure S1.** Mass spectrum of tLyP-1(CGNKRTR). **Figure S2.** NMR spectrum of tLyP-1, PEI, and tLyP-1-PEI. **Figure S3.** HPLC result of tLyP-1 before (upper) and after (lower) conjugating to PEI. **Figure S4.** TOF-MS spectrum of PLGA: calcd. 14955.22 and tLyP-1-PEI-PLGA: calcd. 20722.68. **Figure S5.** Fluorescence image of DiI-labeled TPP@PTX-CuTCPP, the scale bar is 20 μm (inset: optical image of TPP@PTX-CuTCPP, the scale bar is 20 μm). **Figure S6.** Size distribution of TPP@PTX-CuTCPP dispersed in various media including deionized water, PBS buffer, and 1640 containing fetal bovine serum (FBS). **Figure S7.** Digital photography of NPs before (left) and after (right) encapsulation of CuTCPP. **Figure S8.** The standard curve of CuTCPP constructed from UV–vis spectrum at the wavelength of 412 nm. **Figure S9.** The standard curve of PTX constructed from HPLC measurements at the wavelength of 227 nm. **Figure S10.** Intracellular drug retention of MCF-7/Taxol cells treated with DiI-labeled PP@PTX NPs, P@PTX NPs, or free FITC-PTX after replacement with fresh culture medium for another 6 h. The scale bar is 50 μm. **Figure S11.** Bio-TEM images of MCF-7/Taxol cells before (**a**) and after (**b**) coincubation with PP@PTX NPs for 3 h. **Figure S12.** Three-dimensional viewer based on three-dimensional reconstruction of the MCF-7/Taxol spheroid models incubated with DiI-labeled TPP@PTX-CuTCPP for 6 h. **Figure S13.** Three-dimensional viewer based on three-dimensional reconstruction of the MCF-7/Taxol spheroid models incubated with DiI-labeled PP@PTX-CuTCPP for 6 h. **Figure S14.** Quantitative fluorescence intensity of NPs with and without tLyP-1 in tumor tissue and the major organs. Data are presented as mean ± SD. **p < 0.01. **Figure S15.** Ultrathin frozen section of main organs (heart, liver, spleen, lungs, and kidneys) and tumor tissues at 24 h postinjection of DiI-labeled TPP@PTX-CuTCPP NPs or PP@PTX-CuTCPP NPs. Nuclei were stained by DAPI. The scale bar is 50 μm. **Figure S16.** The localization of DiI-labeled PP@PTX-CuTCPP NPs in the tumor tissue. Blue fluorescence represents nuclei stained by DAPI and the green represents microvessels stained by CD31. White arrows show representative NPs accumulating in blood vessels. The scale bar is 50 μm. **Figure S17.** The localization of DiI-labeled TPP@PTX-CuTCPP NPs in the tumor tissue. Blue fluorescence represents nuclei stained by DAPI and the green represents microvessels stained by CD31. The scale bar is 50 μm. **Figure S18.** Atomic percentage analysis constructed from XPS spectrum for the Cu2p regions of CuTCPP before (**a**) and after (**b**) treatment with GSH solution. **Figure S19.** A TEM image of releasing process of TPP@PTX-CuTCPP NPs after treatment with buffer solution at pH 6.3 for 24 h. The scale bar is 0.2 μm. **Figure S20.** Cell viability of MCF-7/Taxol cells after coincubation with various concentrations of TPP@PTX-CuTCPP NPs for 24 h. Data are presented as mean  ± SD, n = 4 per group. **p < 0.01. **Figure S21.** H&E staining of the major organs in mice sacrificed at 16 d after various treatments. The scale bar is 50 μm. Note: G1-G8 represent Saline, Free PTX, P@PTX, P@PTX-CuTCPP, PP@PTX, TPP@PTX, PP@PTX-CuTCPP, and TPP@PTX-CuTCPP, respectively. All data are presented as mean ± SD, *p < 0.05, **p < 0.01. **Figure S22**. Time-related body weight curves of mice after various treatments. Data are presented as mean ± SD, n = 5 per group. **Figure S23.** H&E staining on distant tumor sections of 4T1 tumor-bearing mice after various treatments. The scale bar is 50 μm. **Figure S24.** The amount of tumor-infiltrating leucocyte cells detected by flow cytometry plots in mimic distant tumors of different groups. **Figure S25.** Cytokine levels in serum from mice of different groups after various treatments. Data are presented as mean ± SD, n = 5 per group. **Table S1.** Intracellular GSH level of the MCF-7/Taxol cells after various treatments for 24 h. **Table S2.** Combination Index of Different Reversing Strategies.

## Data Availability

The data are available in the main manuscript, supplementary Information files are available from the corresponding author by request.
