# Supplementary material for: Amplified antitumor efficacy by a targeted drug retention and chemosensitization strategy-based “combo” nanoagent together with PD-L1 blockade in reversing multidrug resistance
Source: J Nanobiotechnology. 2021 Jul 5;19:200. doi: 10.1186/s12951-021-00947-9 (PMC8256488; doi:10.1186/s12951-021-00947-9)
Supplement: Supplementary file 1 — Additional file 1: Scheme S1. The synthetic route of tLyP-1-PEI-PLGA. Figure S1. Mass spectrum of tLyP-1(CGNKRTR). Figure S2. NMR spectrum of tLyP-1, PEI, and tLyP-1-PEI. Figure S3. HPLC result of tLyP-1 before (upper) and after (lower) conjugating to PEI. Figure S4. TOF-MS spectrum of PLGA: calcd. 14955.22 and tLyP-1-PEI-PLGA: calcd. 20722.68. Figure S5. Fluorescence image of DiI-labeled TPP@PTX-CuTCPP, the scale bar is 20 μm (inset: optical image of TPP@PTX-CuTCPP, the scale bar is 20 μm). Figure S6. Size distribution of TPP@PTX-CuTCPP dispersed in various media including deionized water, PBS buffer, and 1640 containing fetal bovine serum (FBS). Figure S7. Digital photography of NPs before (left) and after (right) encapsulation of CuTCPP. Figure S8. The standard curve of CuTCPP constructed from UV–vis spectrum at the wavelength of 412 nm. Figure S9. The standard curve of PTX constructed from HPLC measurements at the wavelength of 227 nm. Figure S10. Intracellular drug retention of MCF-7/Taxol cells treated with DiI-labeled PP@PTX NPs, P@PTX NPs, or free FITC-PTX after replacement with fresh culture medium for another 6 h. The scale bar is 50 μm. Figure S11. Bio-TEM images of MCF-7/Taxol cells before (a) and after (b) coincubation with PP@PTX NPs for 3 h. Figure S12. Three-dimensional viewer based on three-dimensional reconstruction of the MCF-7/Taxol spheroid models incubated with DiI-labeled TPP@PTX-CuTCPP for 6 h. Figure S13. Three-dimensional viewer based on three-dimensional reconstruction of the MCF-7/Taxol spheroid models incubated with DiI-labeled PP@PTX-CuTCPP for 6 h. Figure S14. Quantitative fluorescence intensity of NPs with and without tLyP-1 in tumor tissue and the major organs. Data are presented as mean ± SD. **p < 0.01. Figure S15. Ultrathin frozen section of main organs (heart, liver, spleen, lungs, and kidneys) and tumor tissues at 24 h postinjection of DiI-labeled TPP@PTX-CuTCPP NPs or PP@PTX-CuTCPP NPs. Nuclei were stained by DAPI. The scale ba [file 12951_2021_947_MOESM1_ESM.doc]

**Amplified Antitumor Efficacy by a Targeted Drug Retention and Chemosensitization Strategy-based “Combo” Nanoagent together with PD-L1 blockade in Reversing Multidrug Resistance**

Weixi Jiang ^1^, Lei Su ^1^, Meng Ao ^1^, Xun Guo ^1^, Chen cheng ^1^, Yuanli Luo ^1^, Zhuoyan Xie ^1^, Xingyue Wang ^1^, Junrui Wang ^1^, Shuling Liu ^2^, Yang Cao ^1^, Pan Li ^1^, Zhigang Wang ^1^, Haitao Ran ^1^, Zhiyi Zhou ^3*^ and Jianli Ren ^1*^

^1^ Department of Ultrasound, Chongqing Key Laboratory of Ultrasound Molecular Imaging, The Second Affiliated Hospital of Chongqing Medical University, Chongqing 400010, P. R. China

^2^ Department of Radiology, The Second Affiliated Hospital of Chongqing Medical University,, Chongqing 400010, P. R. China

^3^ Department of General practice of Chongqing General Hospital, University of Chinese Academy of Sciences, Chongqing 401147, P. R. China

Correspondence: renjianli@cqmu.edu. (Jianli Ren), zzy16966@163.com (Zhiyi Zhou)

**Additional Figures**

**
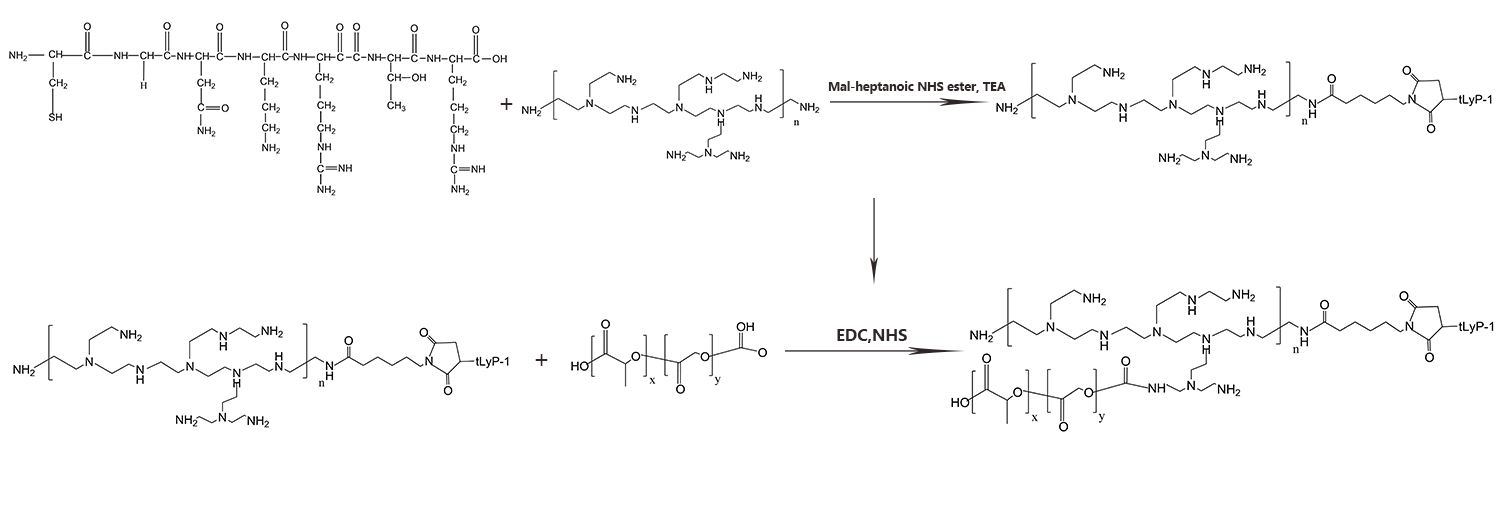
**

**Scheme S1.** The synthetic route of tLyP-1-PEI-PLGA

**
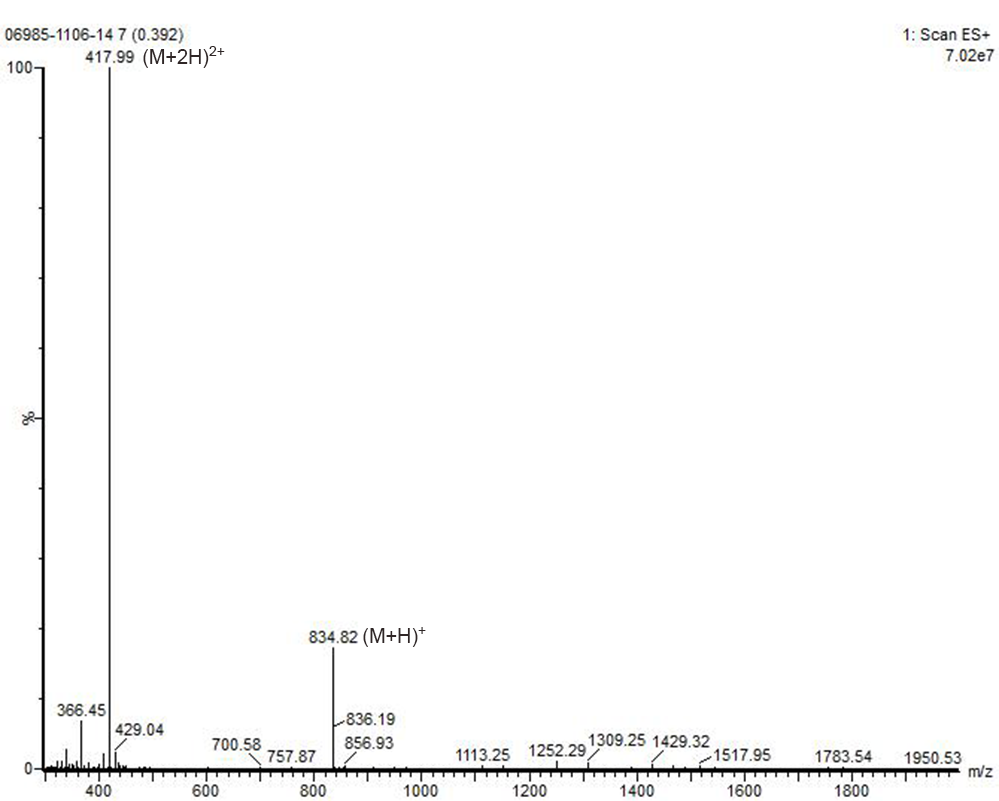
**

**Figure S1.** Mass spectrum of tLyP-1(CGNKRTR).

**
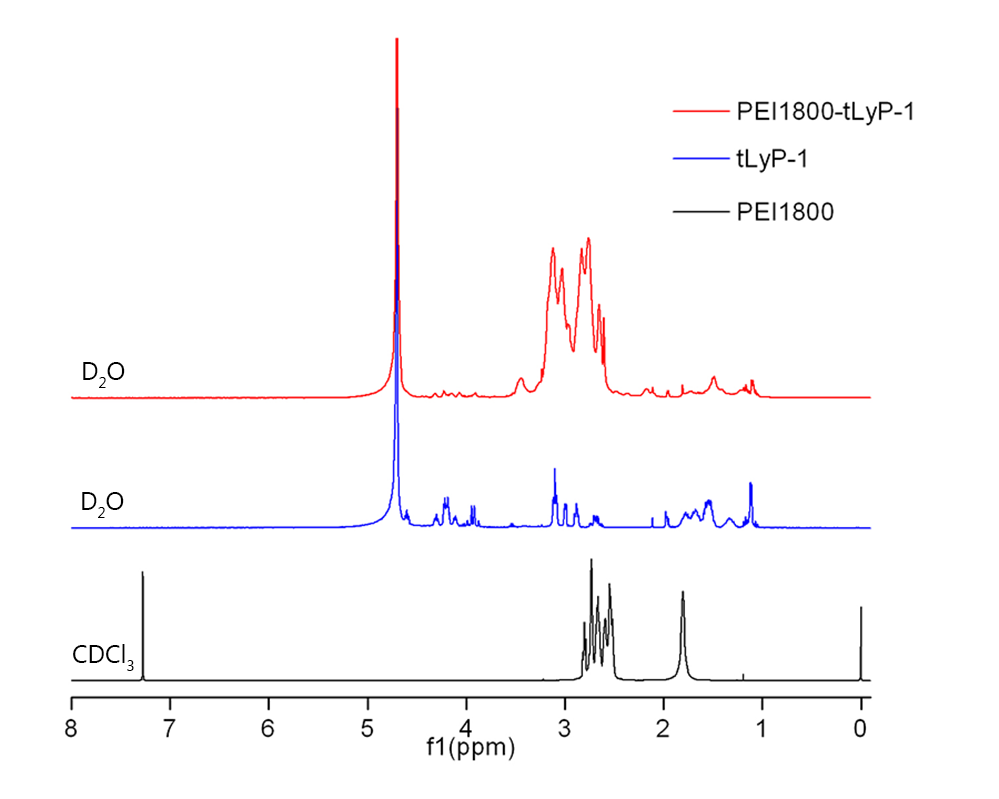
**

**Figure S2.** NMR spectrum of tLyP-1, PEI, and tLyP-1-PEI.

**
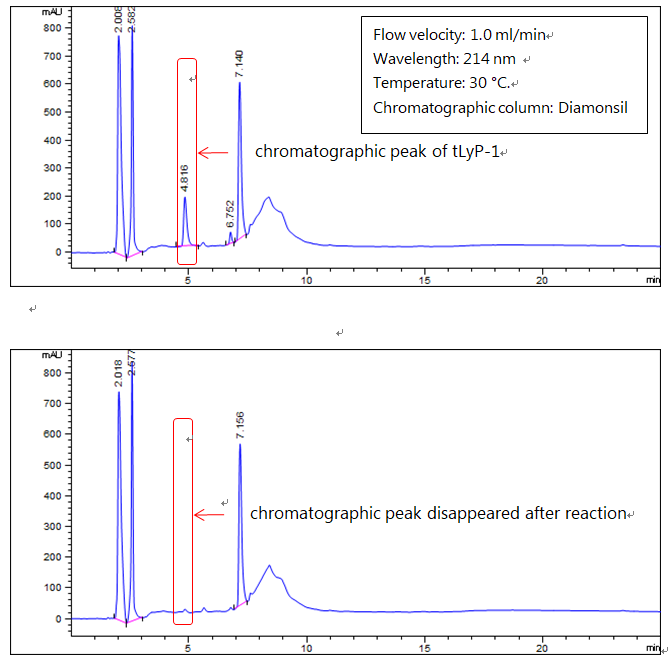
**

**Figure S3.** HPLC result of tLyP-1 before (upper) and after (lower) conjugating to PEI.


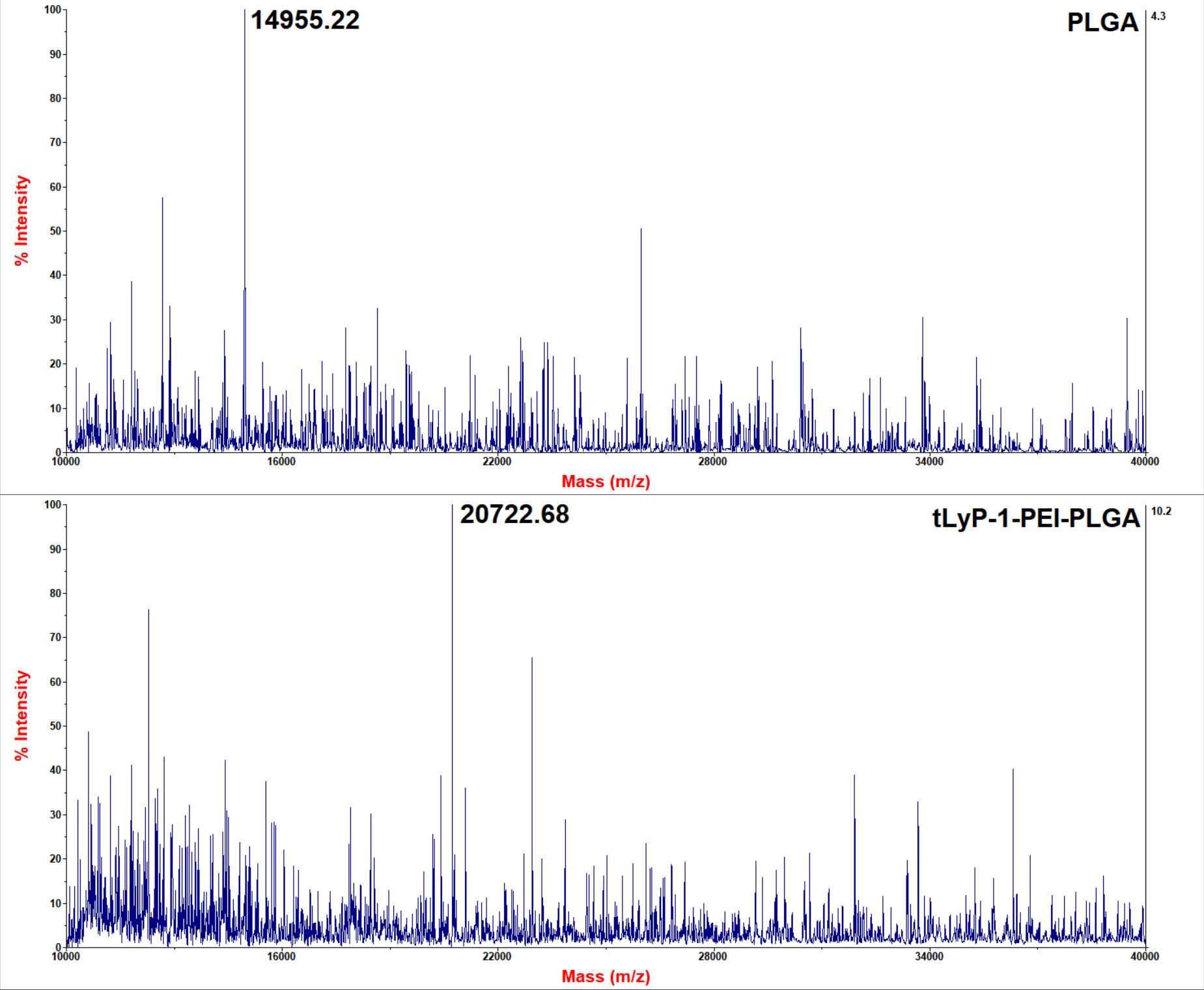


**Figure S4.** TOF-MS spectrum of PLGA: calcd. 14955.22 and tLyP-1-PEI-PLGA: calcd. 20722.68


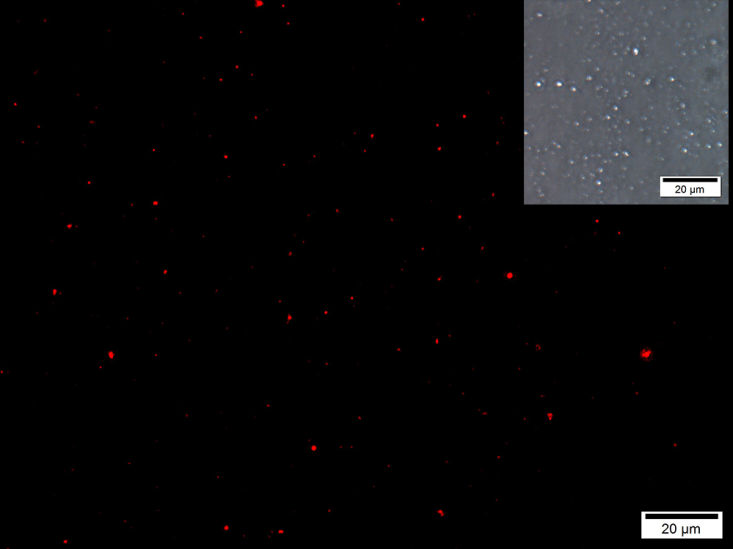


**Figure S5.** Fluorescence image of DiI-labeled TPP@PTX-CuTCPP, the scale bar is 20 μm. (inset: optical image of TPP@PTX-CuTCPP, the scale bar is 20 μm.)

**
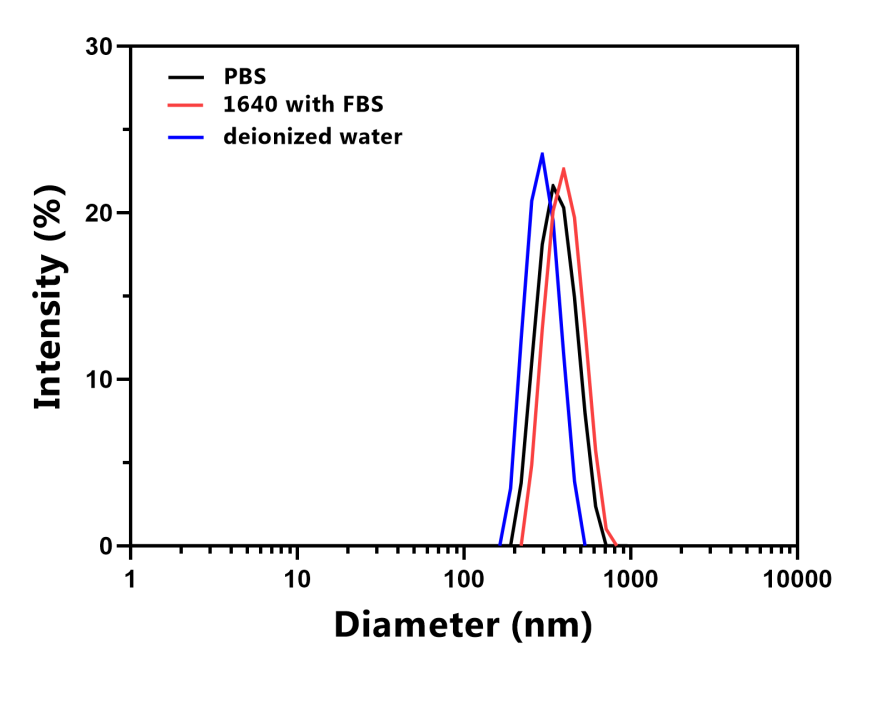
**

**Figure S6.** Size distribution of TPP@PTX-CuTCPP dispersed in various media including deionized water, PBS buffer, and 1640 containing fetal bovine serum (FBS).

**
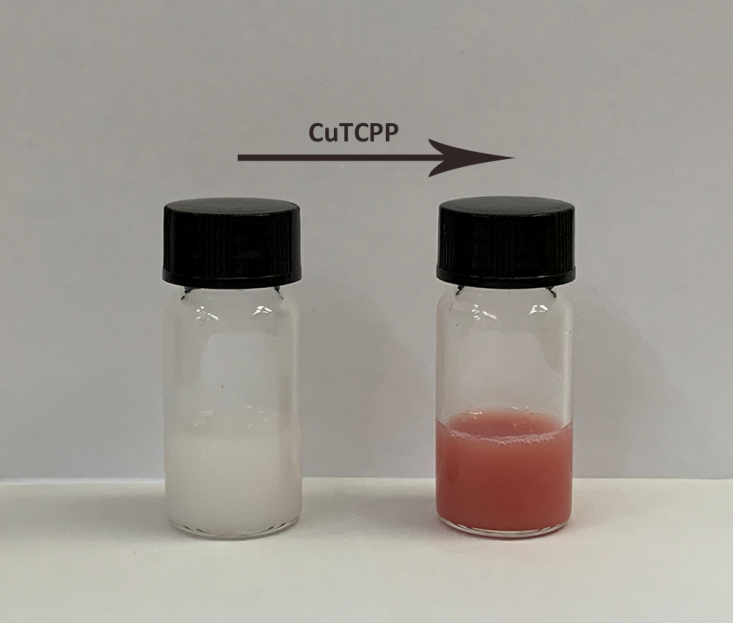
**

**Figure S7.** Digital photography of NPs before (left) and after (right) encapsulation of CuTCPP.

**
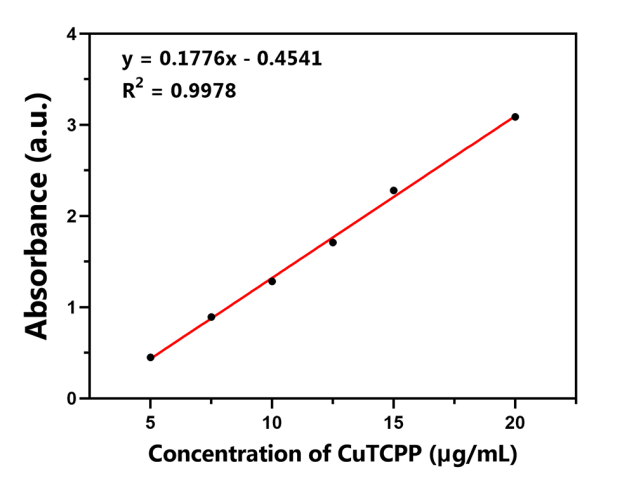
**

**Figure S8.** The standard curve of CuTCPP constructed from UV–vis spectrum at the wavelength of 412 nm.

**
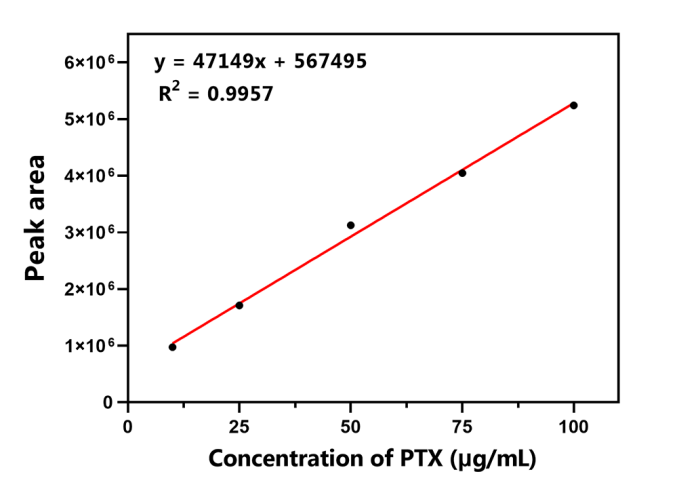
**

**Figure S9.** The standard curve of PTX constructed from HPLC measurements at the wavelength of 227 nm.

**
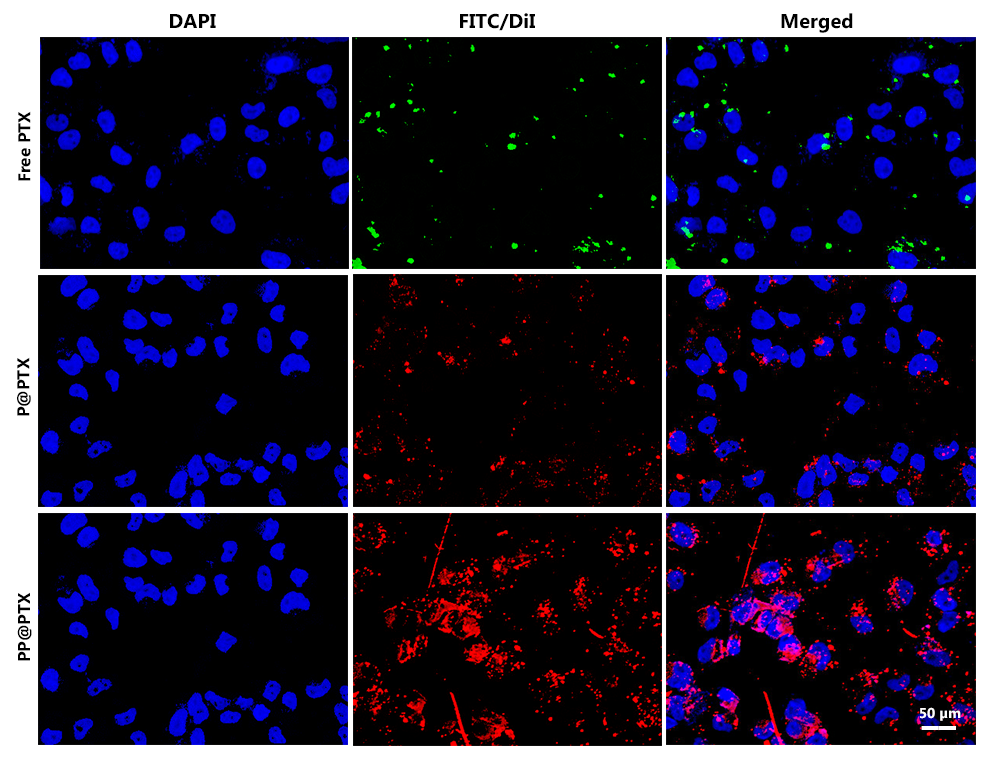
**

**Figure S10.** Intracellular drug retention of MCF-7/Taxol cells treated with DiI-labeled PP@PTX NPs, P@PTX NPs, or free FITC-PTX after replacement with fresh culture medium for another 6 h. The scale bar is 50 μm.


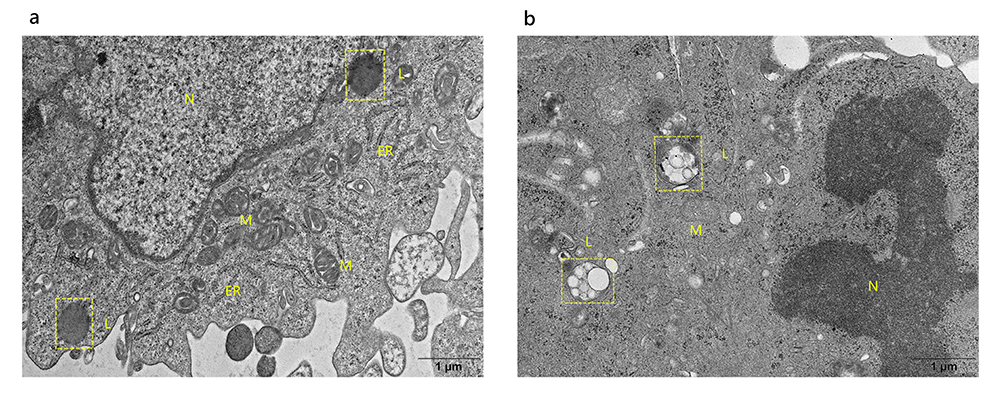


**Figure S11.** Bio-TEM images of MCF-7/Taxol cells before (a) and after (b) coincubation with PP@PTX NPs for 3 h.

**
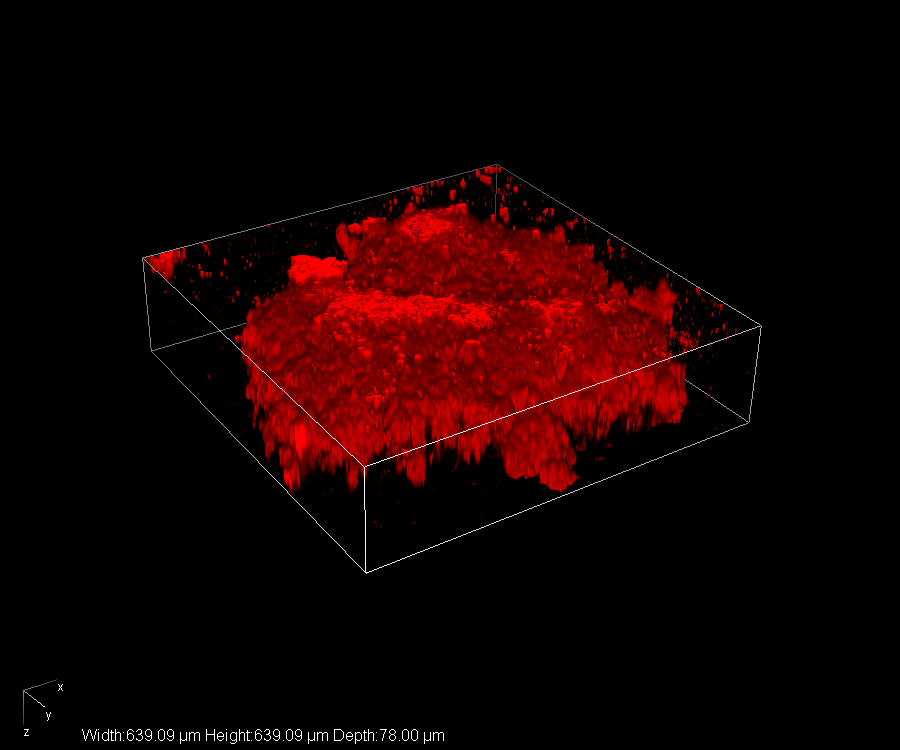
**

**Figure S12.** Three-dimensional viewer based on three-dimensional reconstruction of the MCF-7/Taxol spheroid models incubated with DiI-labeled TPP@PTX-CuTCPP for 6 h.


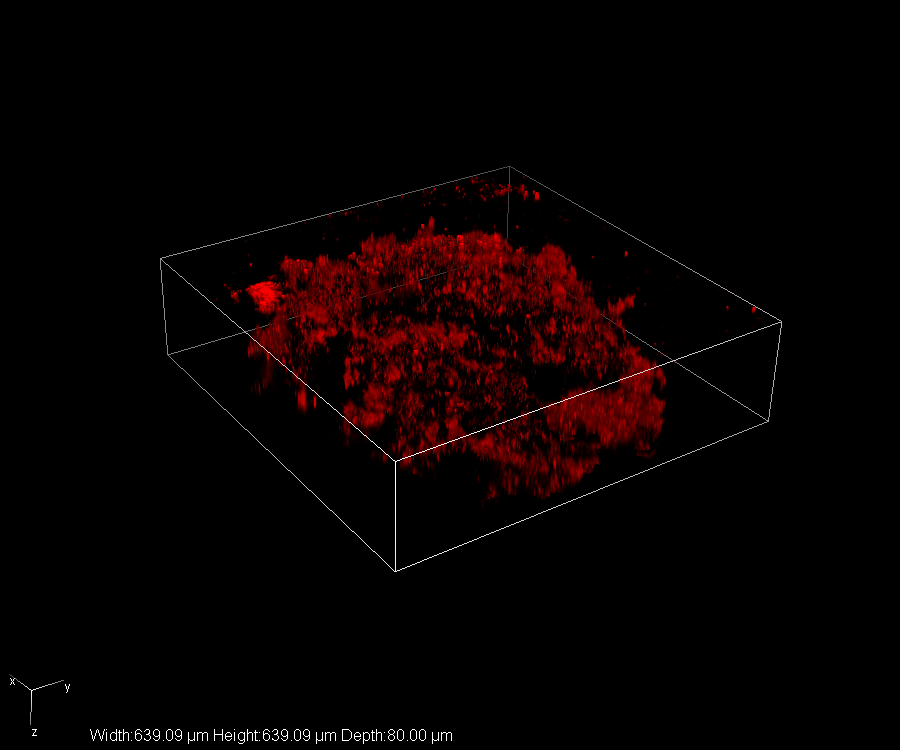


**Figure S13.** Three-dimensional viewer based on three-dimensional reconstruction of the MCF-7/Taxol spheroid models incubated with DiI-labeled PP@PTX-CuTCPP for 6 h.

**
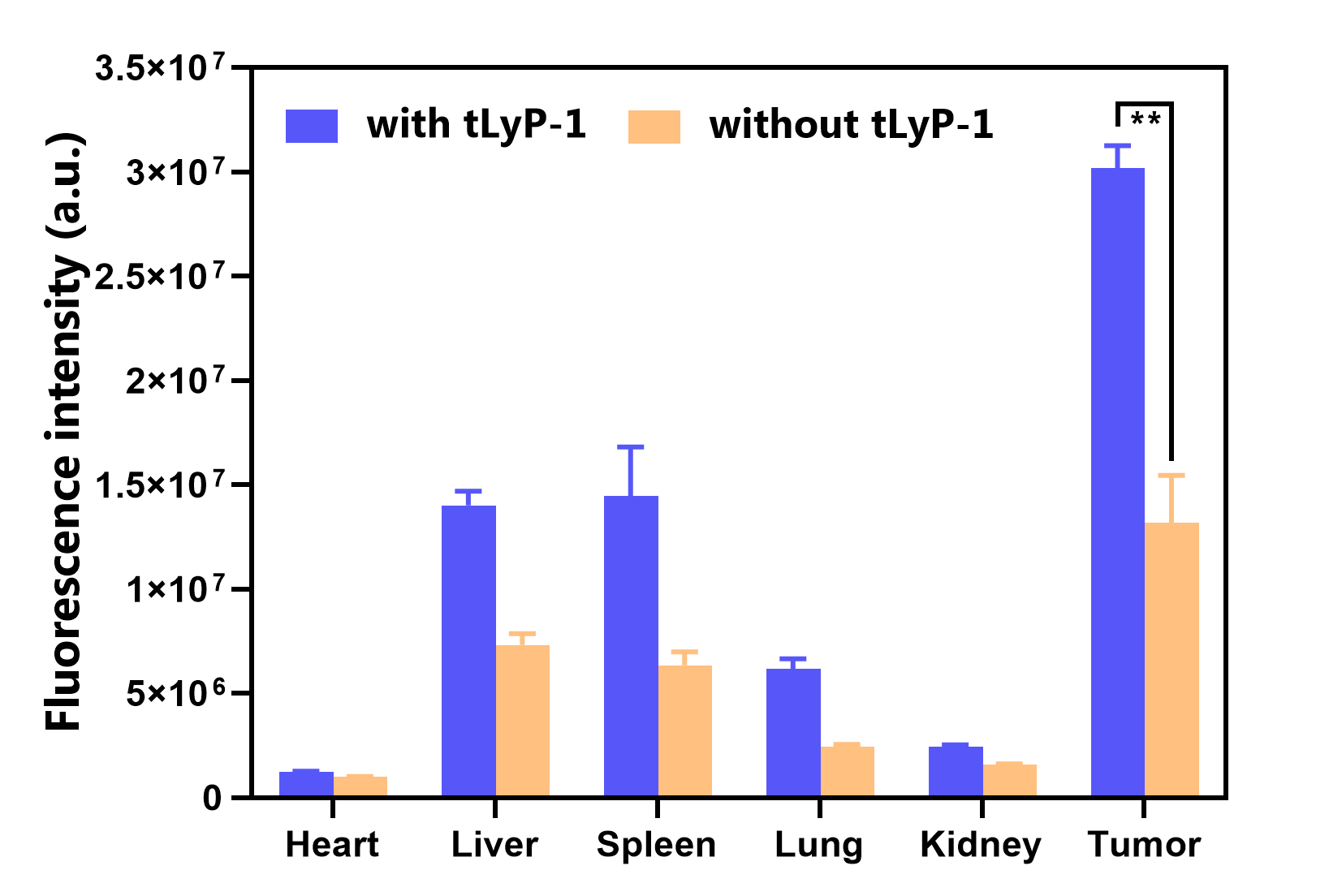
**

**Figure S14.** Quantitative fluorescence intensity of NPs with and without tLyP-1 in tumor tissue and the major organs. Data are presented as mean ± SD. **p < 0.01..

**
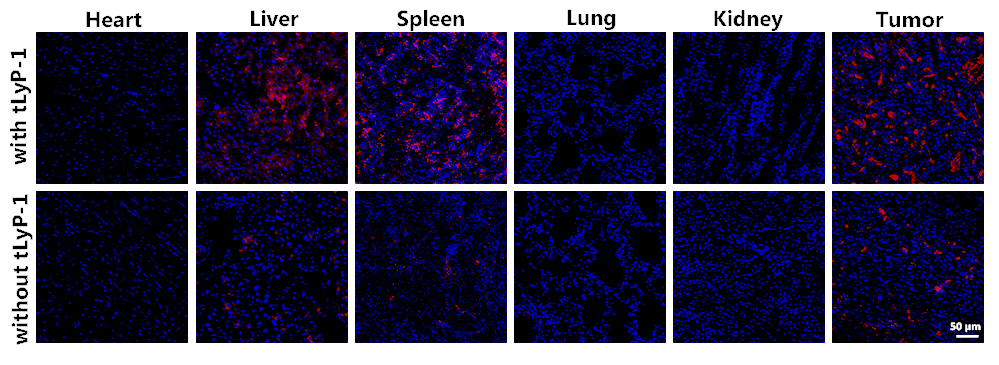
**

**Figure S15.** Ultrathin frozen section of main organs (heart, liver, spleen, lungs, and kidneys) and tumor tissues at 24 h postinjection of DiI-labeled TPP@PTX-CuTCPP NPs or PP@PTX-CuTCPP NPs. Nuclei were stained by DAPI. The scale bar is 50 μm.

**
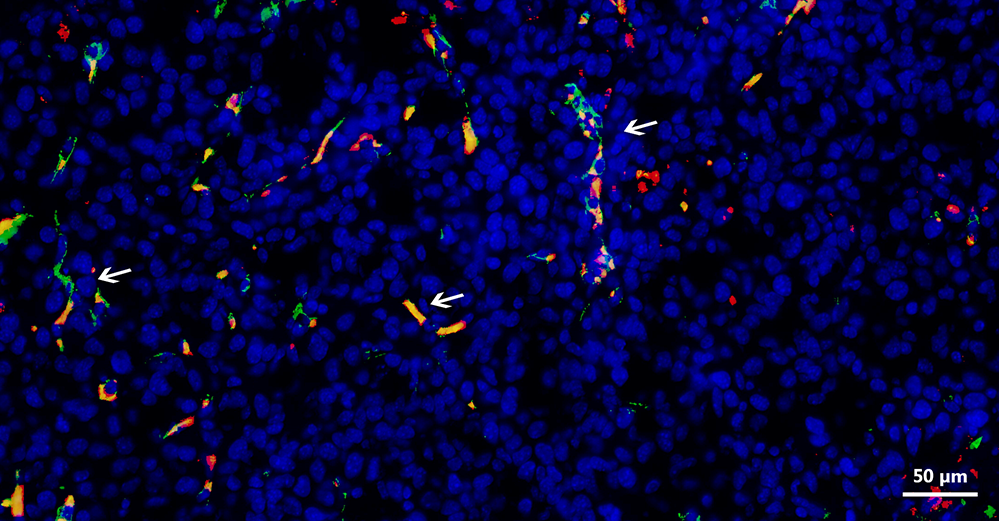
**

**Figure S16.** The localization of DiI-labeled PP@PTX-CuTCPP NPs in the tumor tissue. Blue fluorescence represents nuclei stained by DAPI and the green represents microvessels stained by CD31. White arrows show representative NPs accumulating in blood vessels. The scale bar is 50 μm.


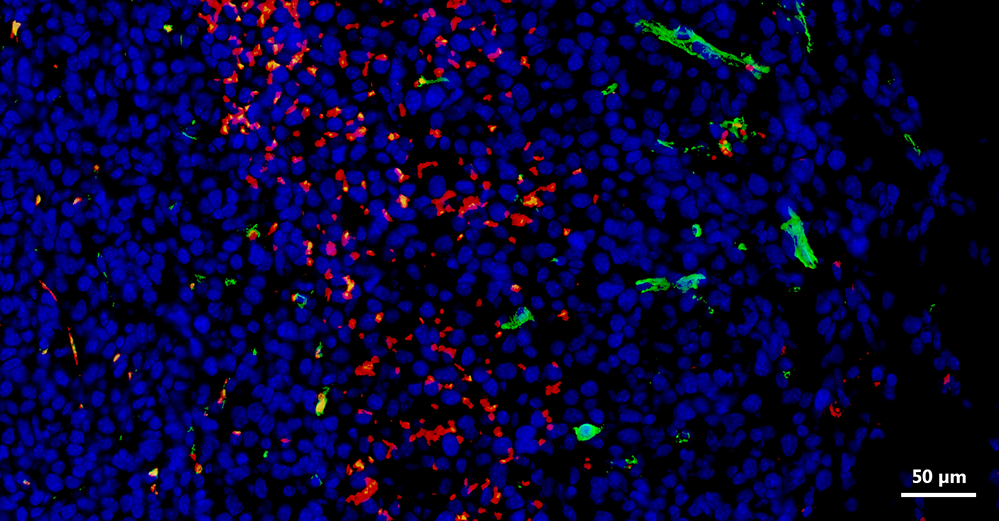


**Figure S17.** The localization of DiI-labeled TPP@PTX-CuTCPP NPs in the tumor tissue. Blue fluorescence represents nuclei stained by DAPI and the green represents microvessels stained by CD31. The scale bar is 50 μm.

**
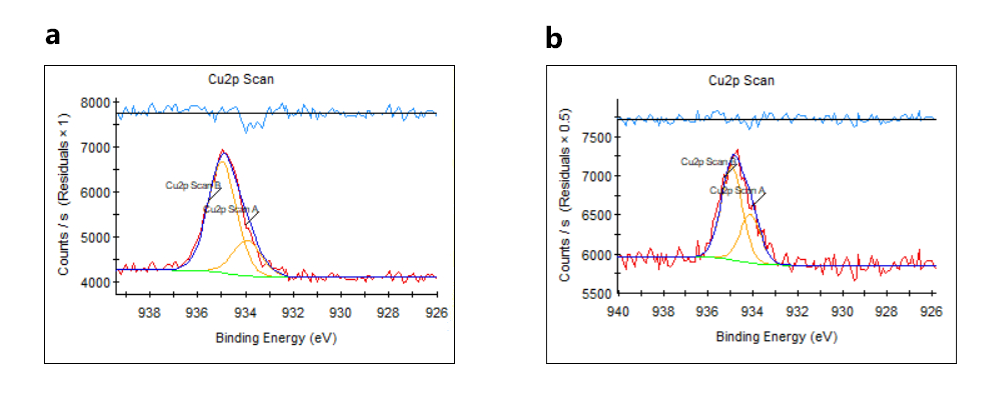
**

**Figure S18.** Atomic percentage analysis constructed from XPS spectrum for the Cu2p regions of CuTCPP before (a) and after (b) treatment with GSH solution.


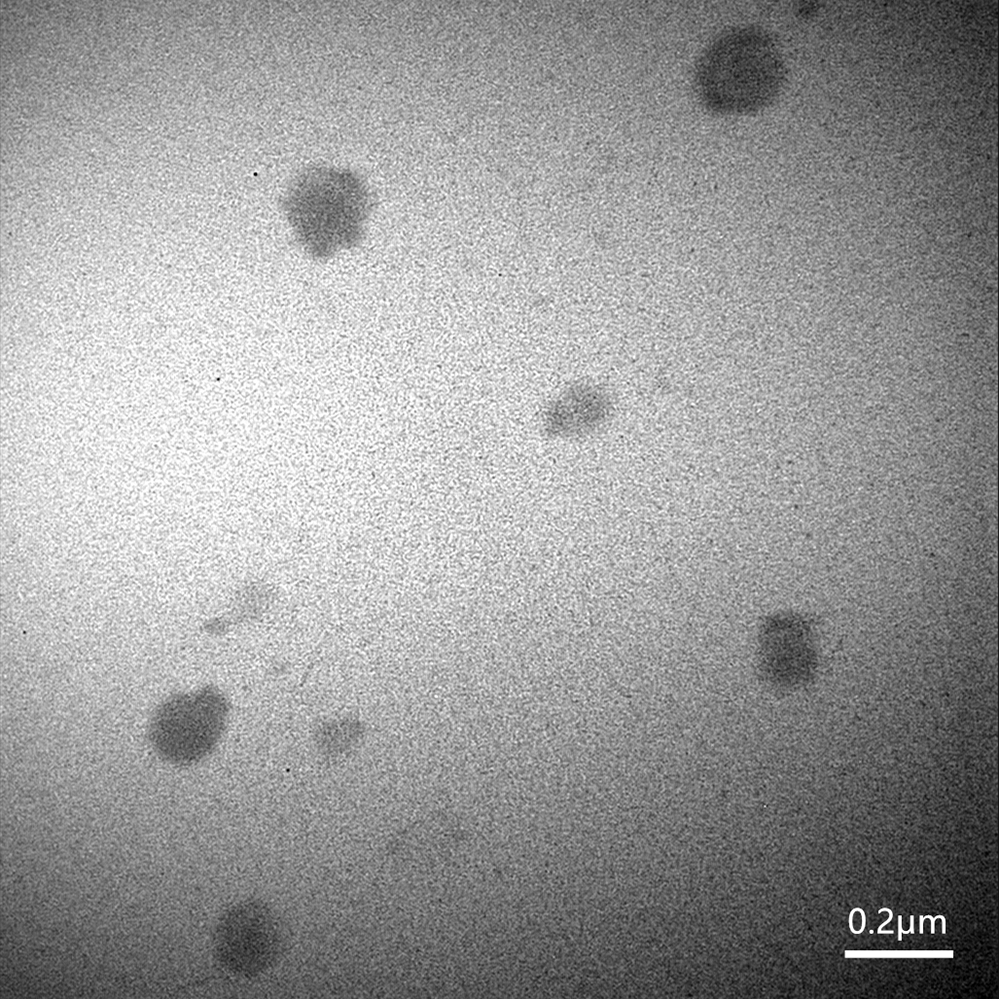


**Figure S19.** A TEM image of releasing process of TPP@PTX-CuTCPP NPs after treatment with buffer solution at pH 6.3 for 24 h. The scale bar is 0.2 μm.


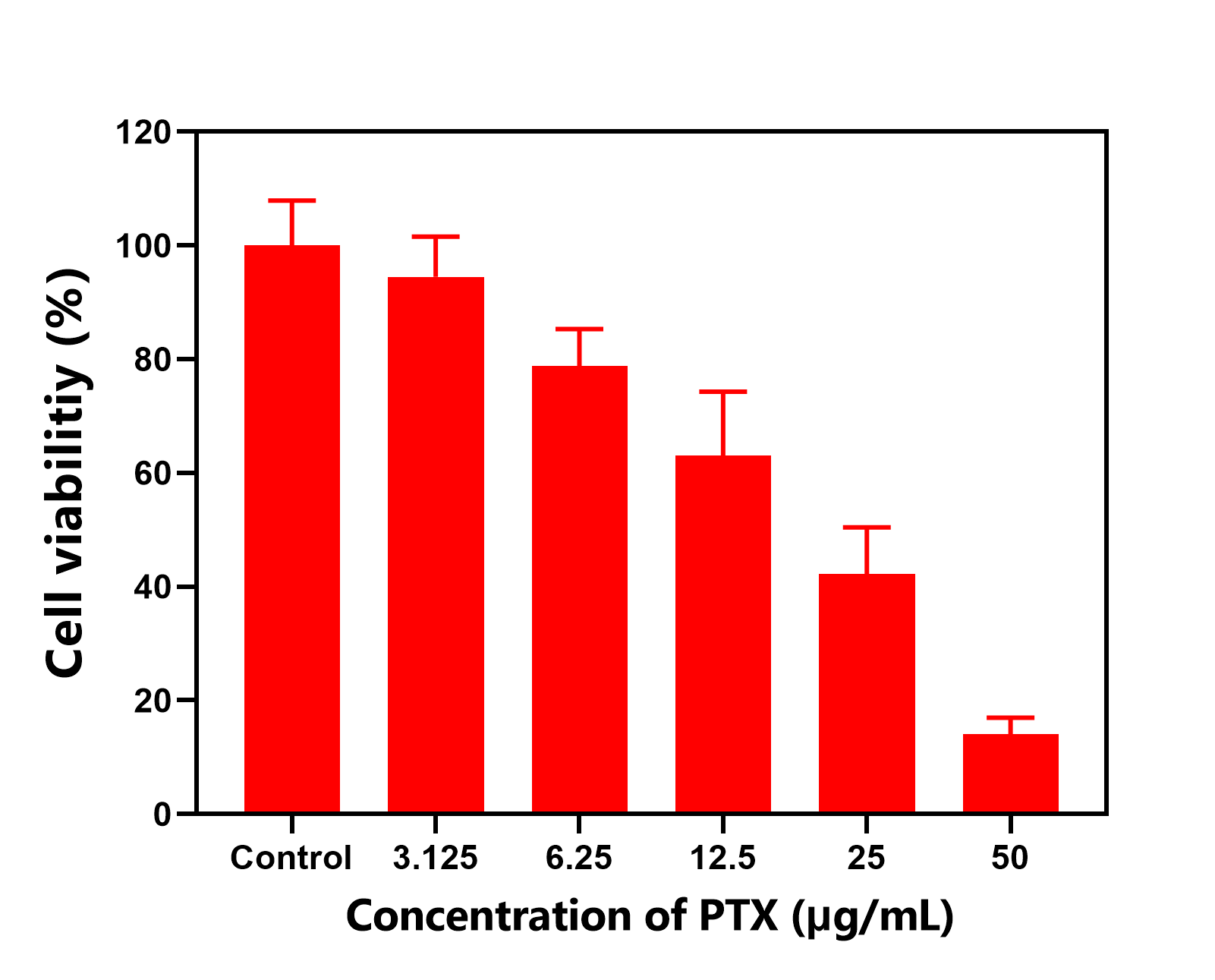


**Figure S20.** Cell viability of MCF-7/Taxol cells after coincubation with various concentrations of TPP@PTX-CuTCPP NPs for 24 h. Data are presented as mean ± SD, n = 4 per group. **p < 0.01.


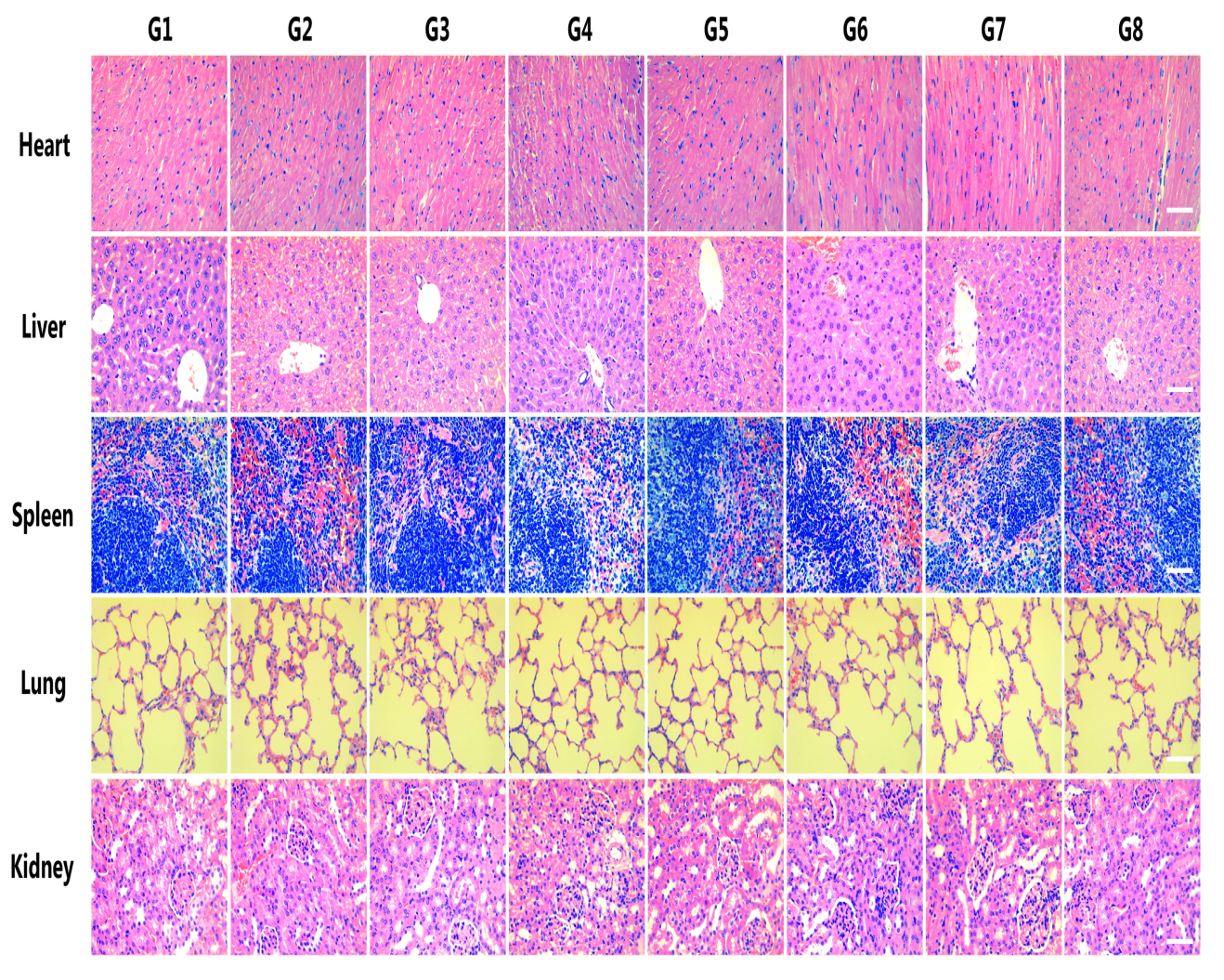


**Figure S21.** H&E staining of the major organs in mice sacrificed at 16 d after various treatments. The scale bar is 50 μm. Note: G1-G8 represent Saline, Free PTX, P@PTX, P@PTX-CuTCPP, PP@PTX, TPP@PTX, PP@PTX-CuTCPP, and TPP@PTX-CuTCPP, respectively. All data are presented as mean ± SD, *p < 0.05, **p < 0.01.


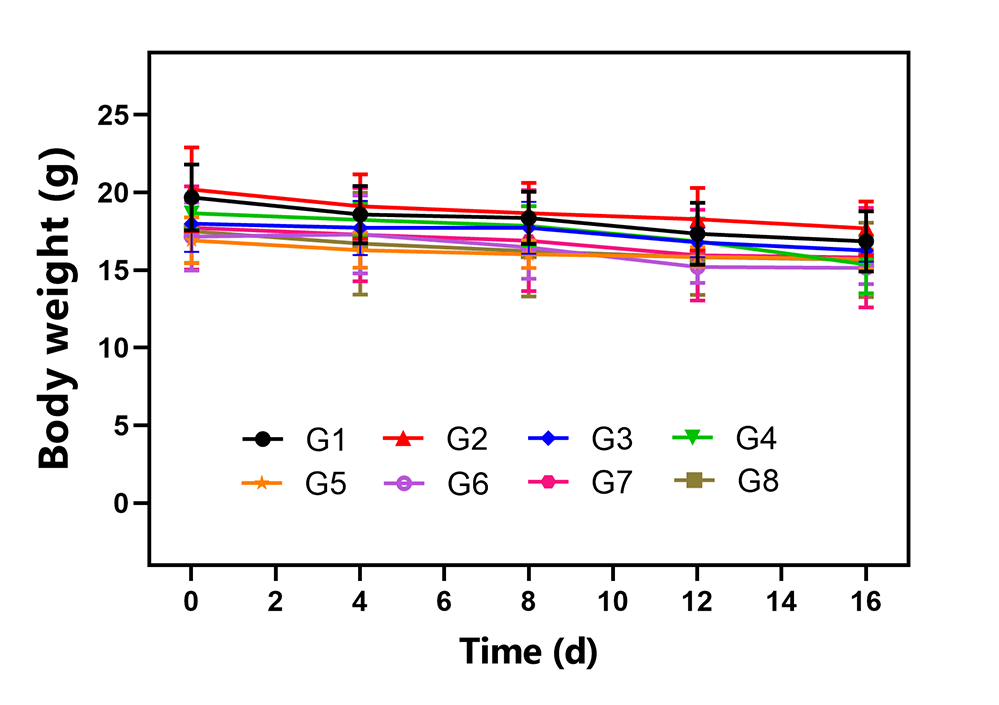


**Figure S22­­.** Time-related body weight curves of mice after various treatments. Data are presented as mean ± SD, n = 5 per group.

**
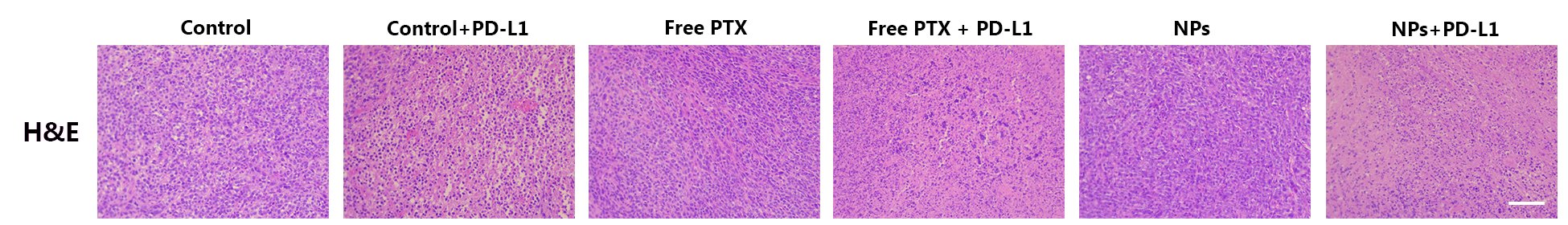
**

**Figure S23.** H&E staining on distant tumor sections of 4T1 tumor-bearing mice after various treatments. The scale bar is 50 μm.

**
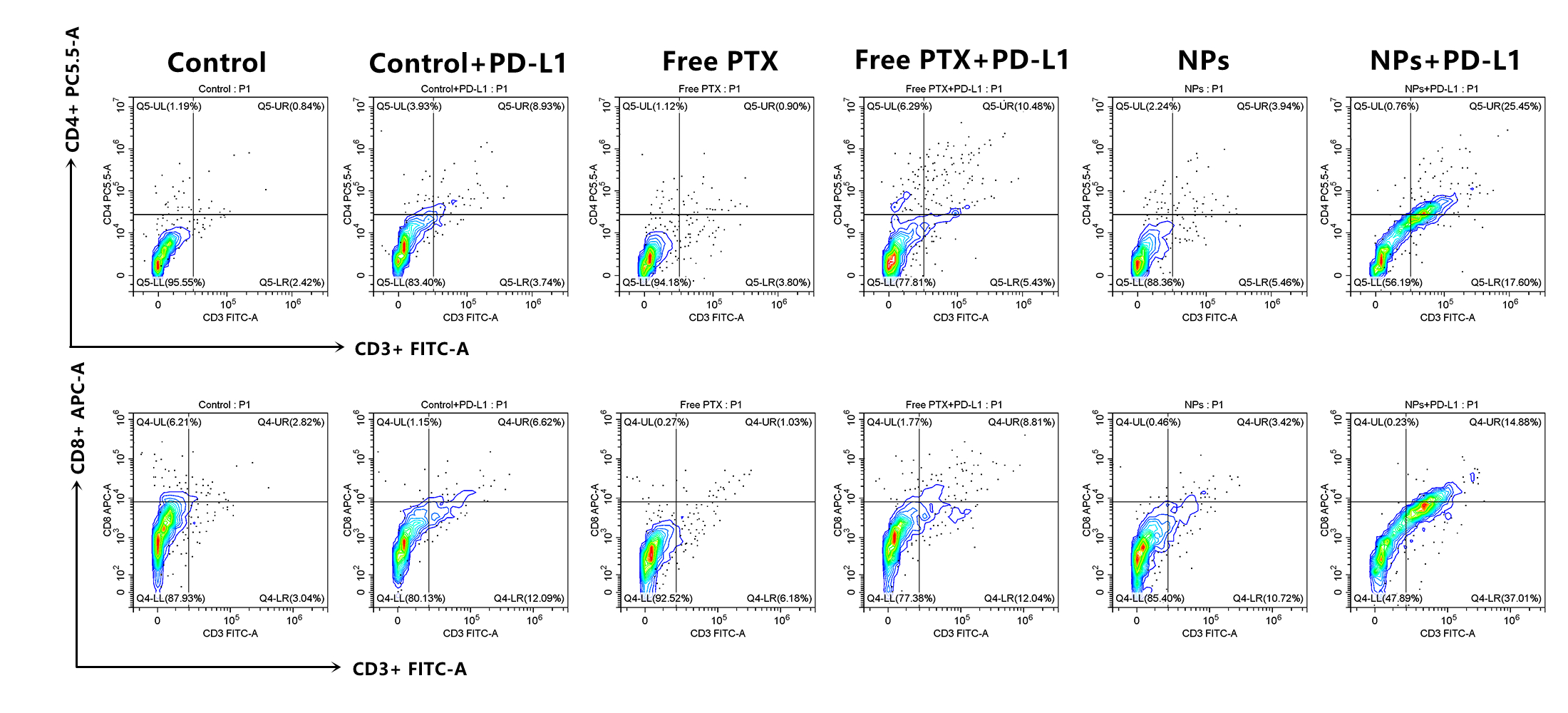
**

**Figure S24.** The amount of tumor-infiltrating leucocyte cells detected by flow cytometry plots in mimic distant tumors of different groups.

**
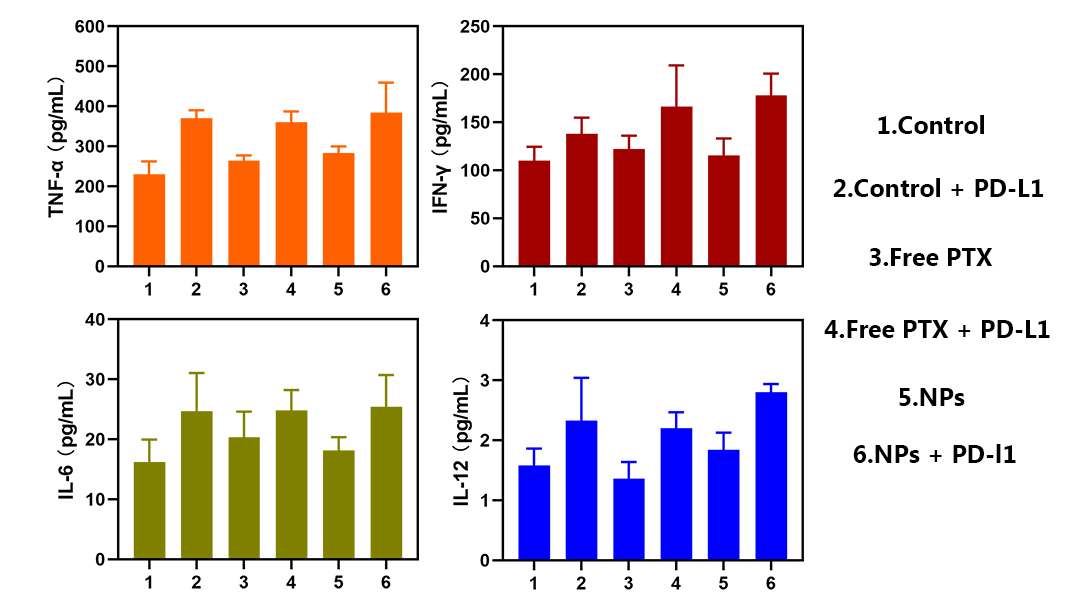
**

**Figure S25** Cytokine levels in serum from mice of different groups after various treatments. Data are presented as mean ± SD, n = 5 per group.

**Additional Table**

**Table S1.** Intracellular GSH level of the MCF-7/Taxol cells after various treatments for 24 h

| Treatment  （24h） | GSH in MCF-7/Taxol  （mmol L^-1^） |
| --- | --- |
| P@CuTCPP | 4.99 ± 0.76 ^**^ |
| PLGA with free BSO ^a^ | 3.05 ± 1.39 ^**^ |
| Untreated | 8.27 ± 0.47 |

^a^ The BSO concentration is 45 μg mL^-1^ for MCF-7/Taxol cells. Data are presented as mean ± SD, n = 5 per group, **p < 0.01.

**Table S2.** Combination Index of Different Reversing Strategies

| Reversing Strategies | Free PTX | PEI + PTX | CuTCPP + PTX | tLyP-1 + PEI + PTX | PEI + CuTCPP + PTX | tLyP-1 + PEI + CuTCPP + PTX |
| --- | --- | --- | --- | --- | --- | --- |
| Combination Index, CI | **—** | 0.927 | 0.896 | 0.800 | 0.498 | 0.201 |
